# GADD45A plays a protective role against temozolomide treatment in glioblastoma cells

**DOI:** 10.1038/s41598-017-06851-3

**Published:** 2017-08-18

**Authors:** Hsiao-Han Wang, Tsuey-Yu Chang, Wei-Chen Lin, Kuo-Chen Wei, Jyh-Wei Shin

**Affiliations:** 1 Institute of Basic Medical Sciences, College of Medicine, National Cheng Kung University, Tainan, Taiwan; 2 Department of Parasitology, College of Medicine, National Cheng Kung University, Tainan, Taiwan; 3 Departments of Neurosurgery, Chang Gung Memorial Hospital, College of Medicine, Chang Gung University, Taoyuan, Taiwan

## Abstract

Glioblastoma multiforme (GBM) is one of the most aggressive cancers. Despite recent advances in multimodal therapies, high-grade glioma remains fatal. Temozolomide (TMZ) is an alkylating agent used worldwide for the clinical treatment of GBM; however, the innate and acquired resistance of GBM limits its application. Here, we found that TMZ inhibited the proliferation and induced the G2/M arrest of GBM cells. Therefore, we performed microarrays to identify the cell cycle- and apoptosis-related genes affected by TMZ. Notably, GADD45A was found to be up-regulated by TMZ in both cell cycle and apoptosis arrays. Furthermore, GADD45A knockdown (GADD45A^kd^) enhanced the cell growth arrest and cell death induced by TMZ, even in natural (T98) and adapted (TR-U373) TMZ-resistant cells. Interestingly, GADD45A^kd^ decreased the expression of O^6^-methylguanine-DNA methyltransferase (MGMT) in TMZ-resistant cells (T98 and TR-U373). In MGMT-deficient/TMZ-sensitive cells (U87 and U373), GADD45A^kd^ decreased TMZ-induced TP53 expression. Thus, in this study, we investigated the genes influenced by TMZ that were important in GBM therapy, and revealed that GADD45A plays a protective role against TMZ treatment which may through TP53-dependent and MGMT-dependent pathway in TMZ-sensitive and TMZ-resistant GBM, respectively. This protective role of GADD45A against TMZ treatment may provide a new therapeutic strategy for GBM treatment.

## Introduction

Glioma is the most common and most aggressive malignant cancer that affects the central nervous system. Clinically, gliomas can be divided into four grades, with grade 4 glioblastoma multiforme (GBM) being the most malignant and deadly. Unfortunately, grade 4 GBM accounts for approximately half of all gliomas^1, 2^. Despite the use of multimodal glioma treatments, GBM continues to present a great therapeutic challenge, and improvements in prognosis remain poor^3^.

The current standard of care for patients with glioma is maximum surgical resection combined with radiotherapy and adjuvant temozolomide (TMZ) treatment. TMZ is a novel oral alkylating agent that damages DNA mainly by methylating the O^6^-position of guanine and causing mismatches with thymine in double-stranded DNA. This mismatch blocks DNA replication, thereby leading to the collapse of replication forks and double-strand breaks and consequently triggering cell death^4^. Furthermore, TMS’s low molecular weight facilitates its movement across the blood brain barrier^5^; therefore, TMZ is considered an efficient chemotherapeutic agent for primary malignant brain tumors^6, 7^. In 2005, TMZ treatment in phase III clinical trials was shown to increase the median survival from 12.1 to 14.6 months and the two-year survival rate from 10 to 26.5%, as compared with postoperative radiotherapy alone in GBM patients^8^. Therefore, TMZ has been well received as a current standard chemotherapeutic agent.

However, despite recent advances in multimodal therapies, the prognosis of GBM remains unsatisfactory. Because GBM patients commonly exhibit resistance to TMZ treatment, the average survival time of GBM patients is still 12–15 months after diagnosis^9, 10^, and no further improvements in outcomes have been recorded since the presentation of radiotherapy-TMZ therapy in 2005^11^. With a better understanding of the changes in the cellular mechanisms during traditional GBM therapy, novel therapeutic targets may be found to optimize therapeutic approaches.

TMZ has been reported to cause cell cycle arrest in the G2/M phase and to mediate apoptosis^12^. The cellular proteins involved in the regulation of the cell cycle and apoptosis are the final arbiters of cell fate under toxicant-induced cell damage^13^. Thus, in the present study, to gain new insights into the mechanisms of cell cycle and apoptosis regulation mediated by TMZ in malignant GBM and to identify new target genes that may provide new therapeutic strategies for TMZ treatment, we sought to identify specific gene expression signatures associated with the cell cycle and apoptosis in response to TMZ treatment by using cDNA microarrays. We identified 5 up-regulated genes/2 down-regulated genes and 5 up-regulated genes/3 down-regulated genes on the cell cycle and apoptosis arrays, respectively, in response to TMZ treatment. Notably, among these genes, GADD45A was found to be up-regulated by TMZ in both the cell cycle and apoptosis arrays in chemo-sensitive U87 cells. Furthermore, GADD45A knockdown (GADD45A^kd^) was accompanied by p21 elevation and enhanced the inhibition of cell growth and increased cell death caused by TMZ treatment even in natural TMZ-resistant GBM (T98) and adapted TMZ-resistant GBM (TR-U373) cells. O^6^-methylguanine-DNA methyltransferase (MGMT) is widely considered to be an indicator of resistance to alkylating agents such as TMZ, and TMZ-induced DNA damage is increased when MGMT expression is abolished^14^. Here, we found that GADD45A^kd^ enhanced the cytotoxic effect of TMZ, and this was accompanied by a decrease in TP53. In addition, GADD45A^kd^ substantially decreased MGMT expression in TMZ-resistant GBM cells. These results revealed that the GADD45A^kd^ induced chemosensitivity of TMZ-resistant cells perhaps via MGMT.

Thus, here, we surveyed the genes affected by TMZ that may be important in GBM therapy. This is the first study to identify that GADD45A plays a protective role against TMZ treatment through TP53-dependent and MGMT-dependent pathway in TMZ-sensitive and TMZ-resistant GBM, respectively.

## Results

### TMZ prolonged the cell cycle of U87 GBM cells

**Figure 1:**
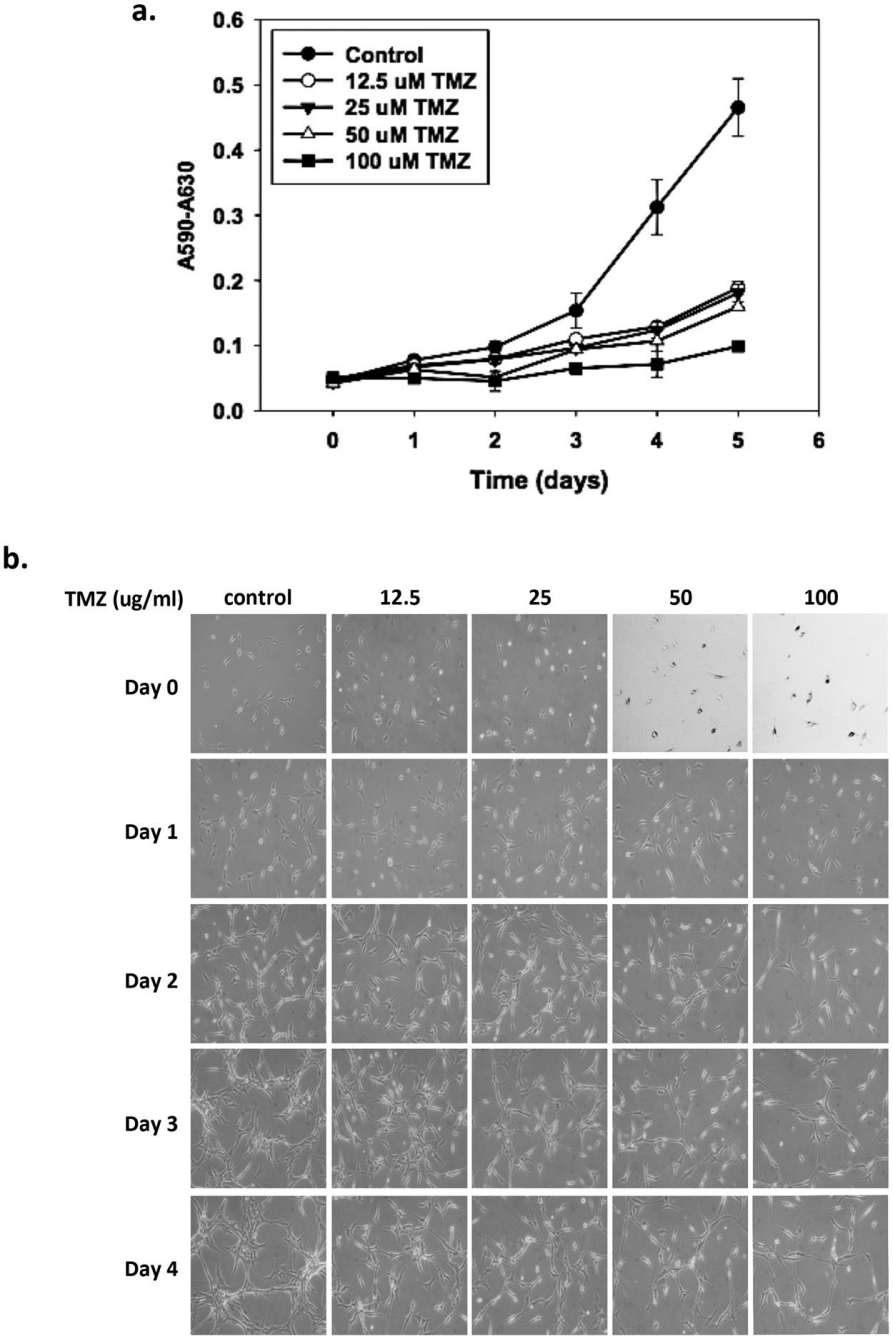
Dose and time-dependent effects on cell proliferation by TMZ in U87 cells. (**a**) The cell viability in various concentrations (–•–, control (DMSO only); –○–, 12.5 μg/ml; –▼–, 25 μg/ml; –△–, 50 μg/ml; –■–, 100 μg/ml) of TMZ treated cells for different time frames: 0 day, 1 day, 2 days, 3 days, 4 days and 5 days. The data were means ± SD of four independent experiments. n = 3 (**b**) Morphology and cell density of U87 glioblastoma cell lines treated with or without vary concentration TMZ (0 μg/ml, control; 12.5 μg/ml; 25 μg/ml; 50 μg/ml; 100 μg/ml) for 0–4 days. Scale bar = 10 μm.

**Figure 2:**
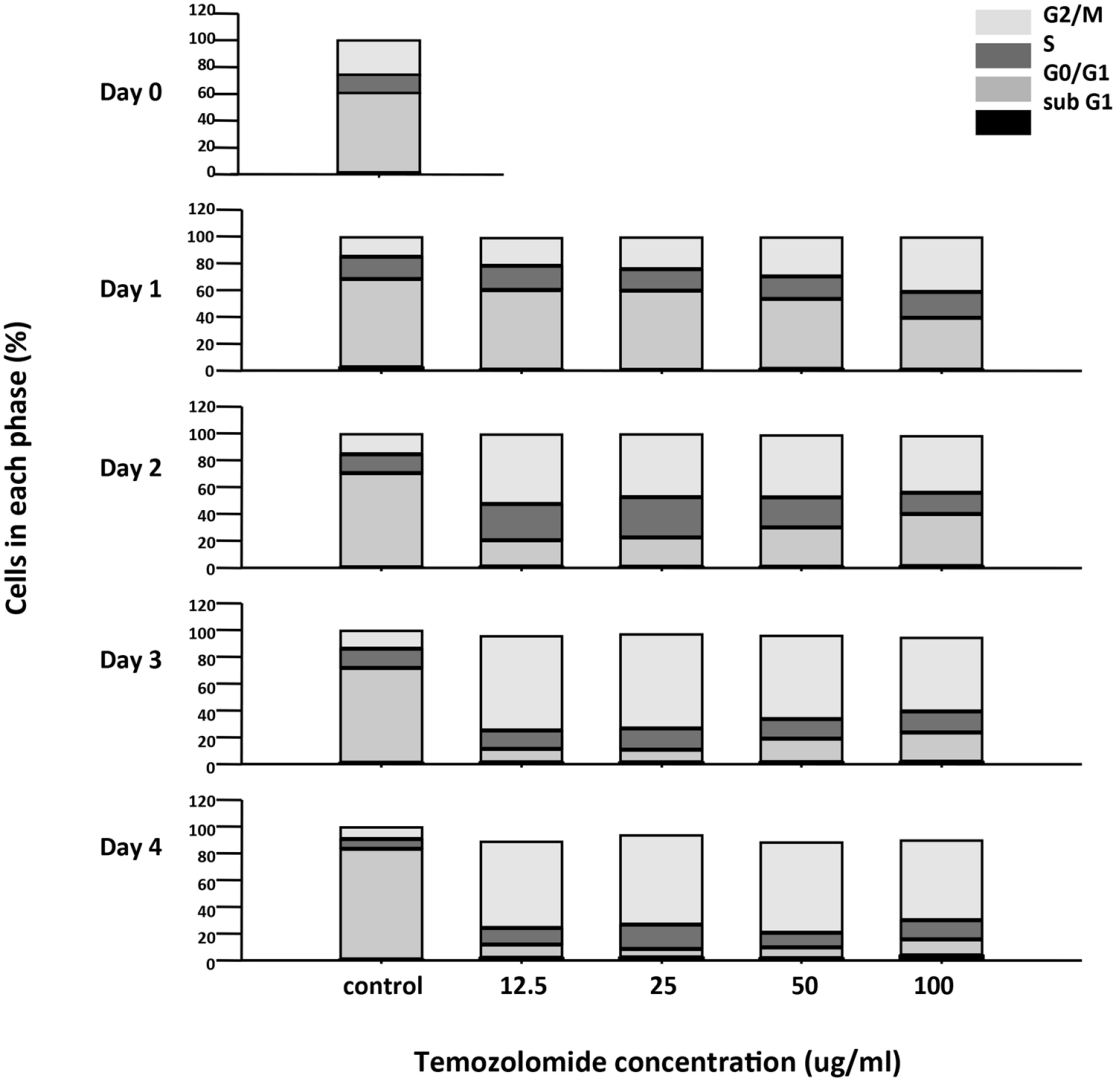
Cell cycle progression after TMZ treatment of human U87 glioblastoma cell lines analysis by FACS. Bar charts illustrating the proportion of cells at different stages in the cell cycle derived from the decomposition of flow cytometry histograms. U87 cells have been treated with vary concentration (0 μg/ml, control; 12.5 μg/ml; 25 μg/ml; 50 μg/ml; 100 μg/ml) TMZ for different time frames: 0 day, 1 day, 2 days, 3 days and 4 days. The U87 cells treated with TMZ were arrest in G2/M stage in dose and time-dependent manner.

To study the effect of TMZ on GBM cell growth and find the optimal experimental conditions, we treated TMZ-sensitive U87 cells with various concentrations (0–100 μg/ml) of TMZ and analyzed cell growth every 24 hours for 120 hours to construct a growth curve by using MTT assays. The results revealed that TMZ decreased the activity of cultured U87 cells in a dose-dependent and time-dependent manner (Fig. 1a). In addition, the cells treated with TMZ had a lower cell density than did the control cells (Fig. 1b). To further confirm the effect of TMZ on cell growth, U87 cells were stained with PI, and the cell cycle was analyzed by flow cytometry. Figure 2 shows that the cell cycle of control cells was G0/G1 dominant, similarly to profiles for other mammalian cells. However, the cell cycle began to arrest in the G2/M phase after 2 days of TMZ treatment at every dosage, without an elevation of the sub-G1 phase. These results confirmed that TMZ inhibited proliferation and caused G2/M arrest of U87 cells. In addition, a significant decrease in the cell number was caused by 25 μg/ml TMZ treatment for 72 and 96 hours (Fig. 1b). Because the U87 cells reached the mid-log phase growth at 96 hours (Fig. 1a), we chose the TMZ concentration of 25 μg/ml and culture time of 96 hours for the experimental condition in the following experiments.

### The effect of TMZ was specific to GBM cells

To determine whether the TMZ-induced anti-tumor effect occurred only in GBM cells, the cell cycles of several human cell lines treated with TMZ were investigated by flow cytometry. TMZ-induced G2/M arrest was observed in only the GBM cell lines U87 and U373 but not in TMZ-resistant T98 cells, HaCaT keratinocytes, Z172 cervical epithelial cells or Hep G2 hepatocellular carcinoma cells (see Supplementary Fig. S1 online). To further confirm the TMZ-resistance of T98 cells, U87 and T98 cells were treated with TMZ, and the cell cycle was analyzed every 24 hours for 7 days. As shown in Supplementary Fig. S1, the proportion of U87 cells in the G2/M phase was substantially increased by TMZ beginning on day 3. However, there was no obvious increase in the G2/M phase of T98 cells after TMZ treatment.

### TMZ affected the expression of several genes that are critical for the cell cycle and apoptosis

**Figure 3:**
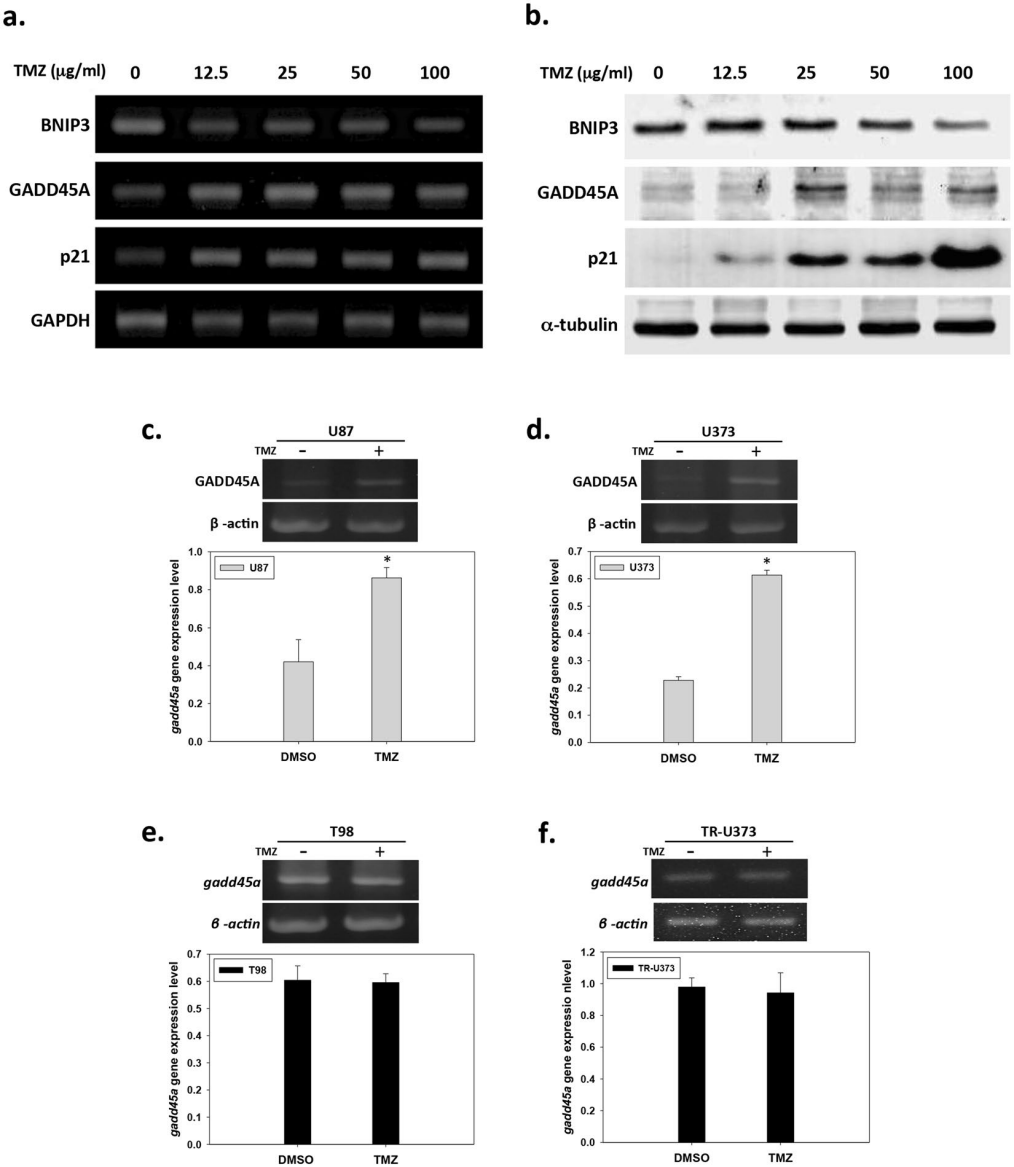
Cell cycle and apoptosis-related genes expression in response to TMZ in human GBM cell lines. (**a**) Analysis of BNIP3, GADD45A and P21 gene expression pattern at fourth day after TMZ treatment in dose-dependent manner (0 μg/ml, control; 12.5 μg/ml; 25 μg/ml; 50 μg/ml; 100 μg/ml) by RT-PCR. (**b**) Analysis of BNIP3, GADD45A and P21 gene expression pattern at fourth day after TMZ treatment in dose-dependent manner (0 μg/ml, control; 12.5 μg/ml; 25 μg/ml; 50 μg/ml; 100 μg/ml) by western blotting. Full-length blot was shown in Supplementary Fig. 5. (**d**),(**e**) Analysis of the expression of GADD45A in U87, U373, TR-U373 and T98 cells at forth day after TMZ (25 μg/ml) treatment by RT-PCR. The bar graph shows the quantification of GADD45A gene expression level in U87 (**c**), U373 (**d**), T98 (**e**) and TR-U373 (**f**) cells. Full-length gels were shown in Supplementary Fig. 3. n = 3, *p < 0.05.

Under toxicant-induced cell damage or stress, the cellular proteins involved in the regulation of the cell cycle and apoptosis are the final arbiters of cell fate^13^. Therefore, we performed a gene expression analysis to identify the cell cycle- and apoptosis-related genes affected by TMZ in TMZ-sensitive U87 and TMZ-resistant T98 cells. The U87 and T98 cells were treated with 25 µg/ml TMZ for 96 hours, and GEArray analyses were performed to analyze the expression of cell cycle- and apoptosis-related genes (see Supplementary Fig. S2). The genes that were substantially up- and down-regulated in response to TMZ in U87 cells are shown in Table 1. Among the 112 gene probes that were analyzed, the results showed that the expression levels of 7 cell cycle-related genes and 8 apoptosis-related genes were substantially changed in U87 cells treated with TMZ. The substantially up-regulated genes included p21, GADD45A, CDK4, PCNA and CCNH, which play important roles in the cell cycle, and TNFRSF10B, GADD45A, TNFSF7, TNFRSF12A and TNFRSF1B, which play roles in apoptosis, were also substantially up-regulated. The cell cycle-associated down-regulated genes were CDC20 and CUL1; the apoptosis-associated down-regulated genes were BNIP3, BID and TNFRSF1A (Table 1). Among these genes, two up-regulated genes (p21 and GADD45A) and one down-regulated gene (BNIP3) that were found to be most affected by TMZ in the microarray analysis were further validated by RT-PCR and western blotting (Fig. 3). The U87 cells were treated with various concentrations of TMZ (0–100 μg/ml). Gene and protein expression levels were detected 96 hours after TMZ treatment. Similarly to the microarray results, TMZ treatment induced dose-dependent down-regulation of BNIP3 at both the transcriptional (Fig. 3a, first panel) and translational levels (Fig. 3b, first panel). On the contrary, the p21 expression was up-regulated by TMZ in dose-dependent manner (Fig. 3a and b, third panel). Similarly to the up-regulation of GADD45A expression that was detected by microarray analysis, GADD45A was also found to be up-regulated by TMZ; however, the highest expression of GADD45A are induced by modest-dose TMZ (25 µg/ml) (Fig. 3a and b, second panel). There were no substantial genetic changes in T98 cells in response to TMZ treatment (see Supplementary Fig. S2 online).

**Table 1:** Cell cycle and apoptosis-related genes expression fold change in U87 GBM cells treated with TMZ.

Gene symbol	Acc. No.	Description	GO Term	Fold change
CDKN1A (p21)	NM_000389	Cyclin-dependent kinase inhibitor 1A (p21, Cip1)	Cell cycle	7.23
GADD45A	NM_001924	Growth arrest and DNA-damage-inducible, alpha	Cell cycle	2.21
TNFRSF10B	NM_003842	Tumor necrosis factor receptor superfamily, member 10b	Apoptosis	2.12
GADD45A	NM_001924	Growth arrest and DNA-damage-inducible, alpha	Apoptosis	1.98
TNFSF7	NM_001252	Tumor necrosis factor (ligand) superfamily, member 7	Apoptosis	1.49
CDK4	NM_000075	Cyclin-dependent kinase 4	Cell cycle	1.33
TNFRSF12A	NM_016639	Tumor necrosis factor receptor superfamily, member 12A	Apoptosis	1.3
PCNA	NM_182649	Proliferating cell nuclear antigen	Cell cycle	1.27
TNFRSF1B	NM_001066	Tumor necrosis factor receptor superfamily, member 1B	Apoptosis	1.26
CCNH	NM_001239	Cyclin H	Cell cycle	1.22
BNIP3	NM_004052	BCL2/adenovirus E1B 19 kDa interacting protein 3	Apoptosis	0.43
CDC20	NM_001255	CDC20 cell division cycle 20 homolog (S. cerevisiae)	Cell cycle	0.8
CUL1	NM_003592	Cullin 1	Cell cycle	0.84
BID	NM_001196	BH3 interacting domain death agonist	Apoptosis	0.85
TNFRSF1A	NM_001065	Tumor necrosis factor receptor superfamily, member 1A	Apoptosis	0.89

Data derived from GEArray^®^ Human cell cycle and apoptosis microarray by hybridizing cDNA synthesized from total mRNA derived from U87 cells treated with or without TMZ (25 μg/ml).

### GADD45A expression increased in both the cell cycle and apoptosis arrays in response to TMZ

We detected several genes affected by TMZ in the U87 GBM cell line, as described above. Notably, GADD45A was found in both the cell cycle and apoptosis arrays to be up-regulated by TMZ in U87 cells. To further confirm the effect of TMZ on GADD45A expression in GBM cells, we treated four different types of GBM cell lines, including two TMZ-sensitive cell lines (U87 and U373), one natural TMZ-resistant cell line (T98) and one adapted TMZ-resistant cell line (TR-U373), with 25 μg/ml TMZ for 96 hours and analyzed GADD45A expression. As shown in Fig. 3, GADD45A expression was elevated by TMZ treatment in U87 and U373 cells (Fig. 3c and d). GADD45A expression was not substantially increased by TMZ in T98 and TR-U373 cells (Fig. 3e and f), although GADD45A expression was higher in TR-U373 cells than in U373 cells.

### Knockdown of GADD45A expression by siRNA

**Figure 4:**
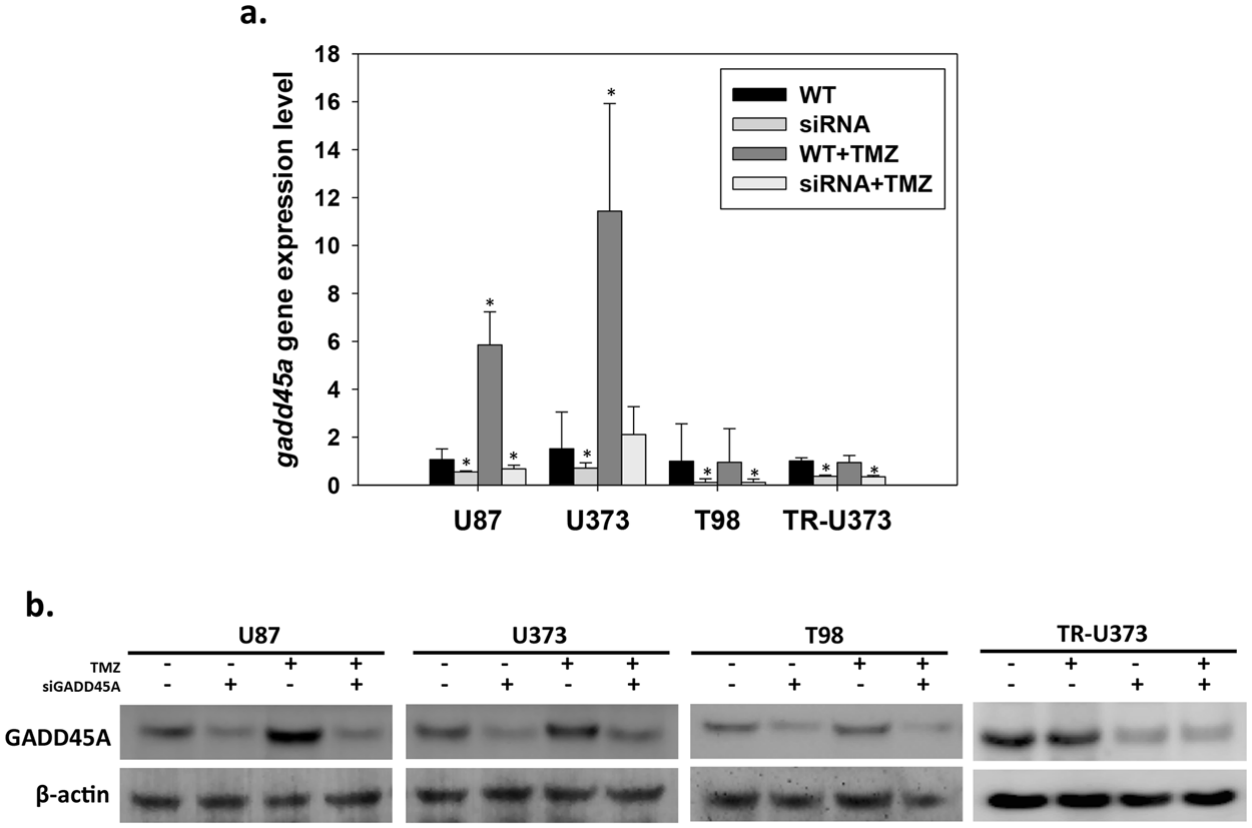
Knockdown GADD45A by specific siRNA in U87, U373, TR-U373 and T98 cells. The cells were pretreated with or without GADD45A siRNA before TMZ (25 μg/ml) or DMSO only treatment. The cells were collected for RNA or protein extraction at day 4 after drug treatment. (**a**) Analysis of GADD45A mRNA expression in U87, U373, TR-U373 and T98 cell lines by qPCR. n = 3, *p < 0.05 (**b**) The expression of GADD45A protein in U87, U373, T98 and TR-U373 cell lines was analyzed by western blotting. Original blot was shown in Supplementary Fig. 7.

To study the effects of GADD45A on cell proliferation and apoptosis after treatment with TMZ, we used siRNA to selectively decrease GADD45A expression in different human GBM cell lines (U87, U373, T98 and TR-U373). qPCR analysis revealed that GADD45A gene expression was decreased by 50% in U87si, U373si, and TR-U373si cells and by 80% in T98si cells (Fig. 4a). Protein expression was analyzed via western blotting, and the results revealed a substantial decrease in GADD45A protein levels in U87si, U373si, T98si and TR-U373si cells (Fig. 4b). To further confirm the efficiency of GADD45A^kd^, GADD45A expression was investigated at both the transcriptional and translational levels after treatment with 25 μg/ml TMZ. As shown in Fig. 4, GADD45A expression was elevated by TMZ treatment in TMZ-sensitive cell lines (U87 and U373); however, it was not substantially increased by TMZ after GADD45A^kd^. These data indicated that, using this approach, we successfully decreased GADD45A expression in all of these GBM cell lines.

### GADD45A^kd^ inhibited cell proliferation

**Figure 5:**
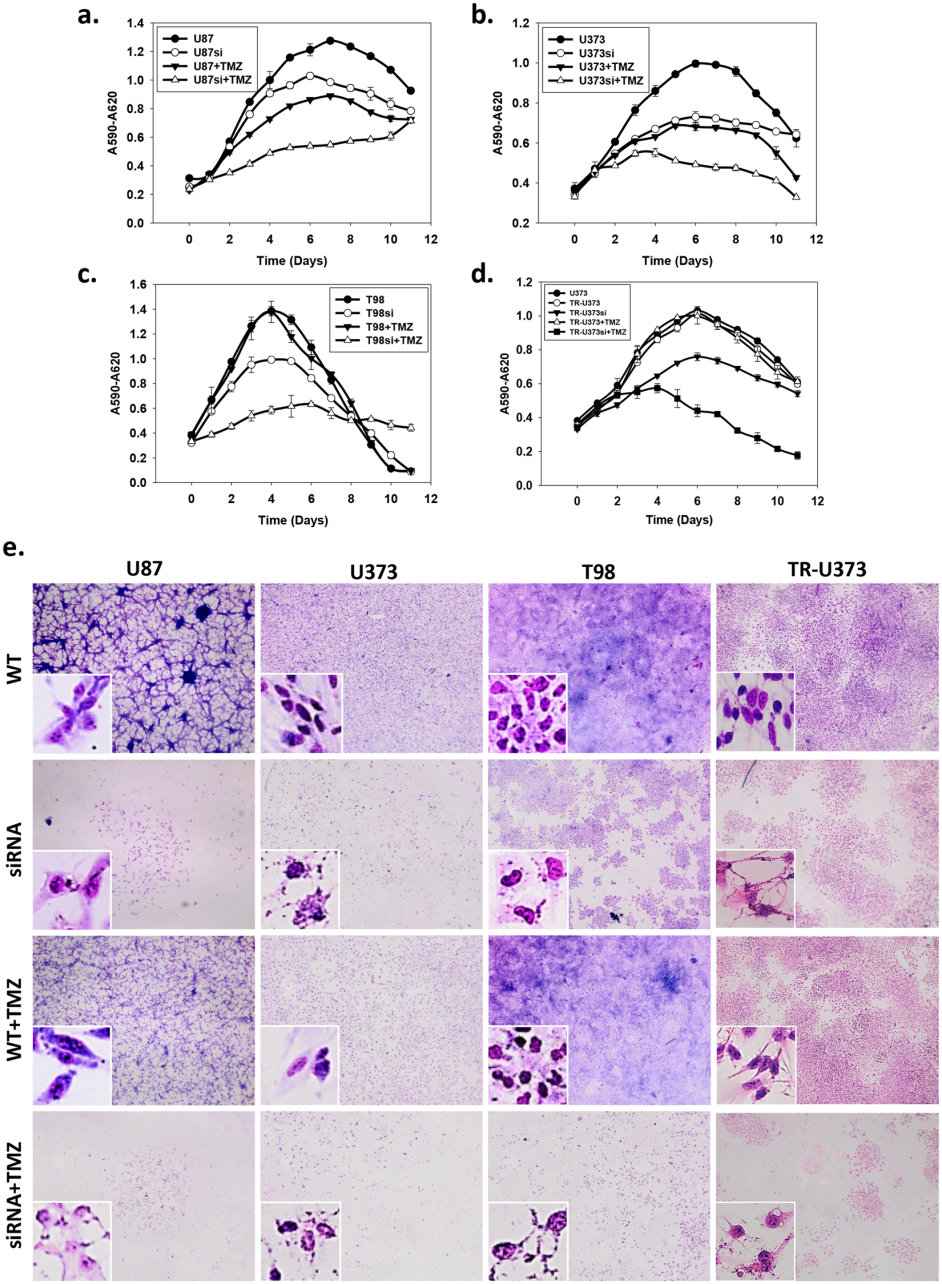
Cell growth and morphology of U87, U373, TR-U373 and T98 cell lines with TMZ or GADD45A siRNA treatment. The cell viability in various conditions (DMSO only –•–, control; GADD45A siRNA treatment –○–; 25 μg/ml TMZ treatment –▼–; combine GADD45A siRNA with 25 μg/ml TMZ treatment –△–) analyzed by MTT assay for 0 to 10 days. (**a**) The cell viability of U87 cells. (**b**) The cell viability of U373 cells. (**c**) The cell viability of T98 cells. (**d**) The cell viability of TR-U373 cells. The data were means ± SD of five independent experiments. n = 3 (**e**) Cells were seeded in 24 well culture plate. After 24 hours, the cells were pretreated with or without GADD45A siRNA before TMZ (25 μg/ml) or DMSO only treatment. The cell morphology and density were observed at day 4 after drug treatment by Giemsa stain. The high magnification view in white frame showed the cell morphology of U87, U373, T98 and TR-U373 cells. Scale bar = 200 μm.

To investigate the effects of GADD45A on cell growth, we assessed cellular activity every 24 hours for 264 hours and constructed a growth curve in different human GBM cell lines, including U87, U373, T98 and TR-U373, by using MTT assays. In order to establish the stable TMZ-resistant cells, the TR-U373 cell was used when it’s growth pattern approximate to normal U373 even under TMZ treatment (Fig. 5d). Similarly to the results described above, TMZ treatment inhibited the proliferation of U87 and U373 cells. The fold changes of the cells with the highest activity levels were 0.69 and 0.68 in U87 and U373 cells, respectively, after TMZ treatment (Fig. 5a and b), whereas this phenomenon was not observed in T98 and TR-U373 cells (Fig. 5c and d). Interestingly, GADD45A^kd^ decreased the proliferation rate of all these cell lines. Moreover, TMZ treatment greatly inhibited proliferation after GADD45A^kd^; the fold changes in the cells with the highest activity levels were decreased to 0.42 and 0.49 in U87si and U373si cells, respectively, after TMZ treatment. Furthermore, a substantial decrease in cellular activity by TMZ was also observed in T98si and TR-U373si cells.

Similar results were obtained on the basis of the cell density, as analyzed by Giemsa staining, showing that the cell number was decreased by TMZ in U87 and U373 cells (Fig. 5e, panels 1 and 2) but not in TR-U373 and T98 cells (Fig. 5e, panels 3 and 4). However, GADD45A^kd^ substantially decreased the cell density in all cell lines. Compared with GADD45A^kd^ alone, the cell density showed a much more severe decrease by GADD45A^kd^ combined with TMZ treatment in all of the GBM cell lines. During the early process of apoptosis, the major morphological changes include cell shrinkage and chromatin condensation. Later on, the nucleus progressively condenses and breaks up accompanied with irregular outlines and form extensions^15^. Under the TMZ treatment only, the morphology of U87 and U373 cells showed early phase of apoptosis with condensed chromatin. The GADD45A^kd^ caused irregular cell outline, cellular fragmentation, condensed chromatin and cell swelling in all of GBM cell lines. Furthermore, this fragmental morphology of cell and nucleus was exacerbated by GADD45A^kd^ combined with TMZ treatment in all of the GBM cell lines (Fig. 5e). These results indicated that GADD45A^kd^ may cause cell apoptosis and enhance the cytotoxicity of TMZ.

### GADD45A^kd^ increased chemo-sensitivity and enhanced TMZ-induced apoptosis

**Figure 6:**
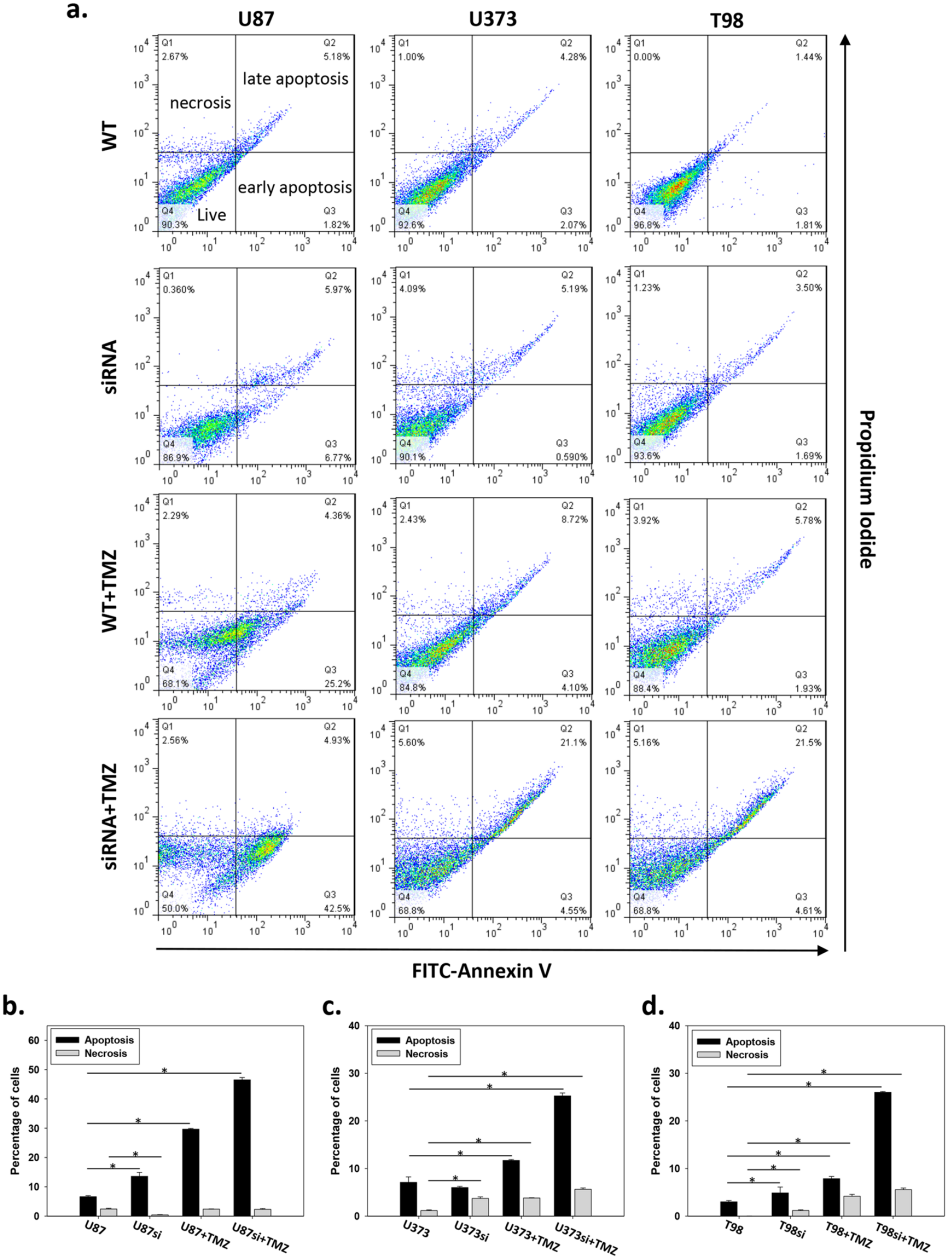
TMZ induced apoptosis in U87, U373 and T98 cell lines with TMZ or GADD45A siRNA treatment. U87, U373 and T98 cells were pretreated with or without GADD45A siRNA before TMZ (25 μg/ml) or DMSO only treatment. The cell apoptosis was examined at day 4 after drug treatment by V-FITC/PI staining and flow cytometry analysis. (**a**) The dot plots shows the percentage of different apoptosis stage. FITC^−^/PI^−^, FITC^+^/PI^−^, FITC^+^/PI^+^, FITC^−^/PI^+^ was regarded as living, early apoptotic, late apoptotic and necrotic cells, respectively. The quantification of percentage of apoptotic (early + late apoptotic cells) and necrotic cells in U87 (**b**), U373 (**c**) and T98 (**d**). n = 3, *p < 0.05.

During apoptosis, the nucleus condenses and segregates into several fragments, and this is followed by disintegration of the cell into apoptotic bodies^15^. As described above, our data revealed that GADD45A^kd^ caused cell morphology to become irregular and fragmented. This phenotype was similar to that of apoptotic cells. Therefore, we investigated whether GADD45A^kd^ induced apoptosis. Using flow cytometric analysis with annexin V/PI double staining, apoptosis was examined in all cell lines after 96 hours of incubation with TMZ (Fig. 6). Apoptosis was substantially elevated by TMZ treatment in U87 (4.49-fold, 29.67% ± 0.24%) and U373 (1.67-fold, 11.7% ± 0.61%) cells when compared to control, but this phenomenon was not found in T98 cells. However, TMZ treatment substantially induced apoptosis in GADD45A-siRNA transfectants of all cell lines, including the TMZ-resistant T98 cell line. Compared with the controls, the apoptosis rates of the GADD45A-siRNA transfectants treated with TMZ were increased 7-fold (46.51% ± 0.79%) in U87 cells, 3.6-fold (25.25% ± 0.6%) in U373 cells, and 8.6-fold (25.99% ± 0.18%) in T98 cells (Fig. 6b,c and d).

### GADD45A^kd^ increased p21 expression

**Figure 7:**
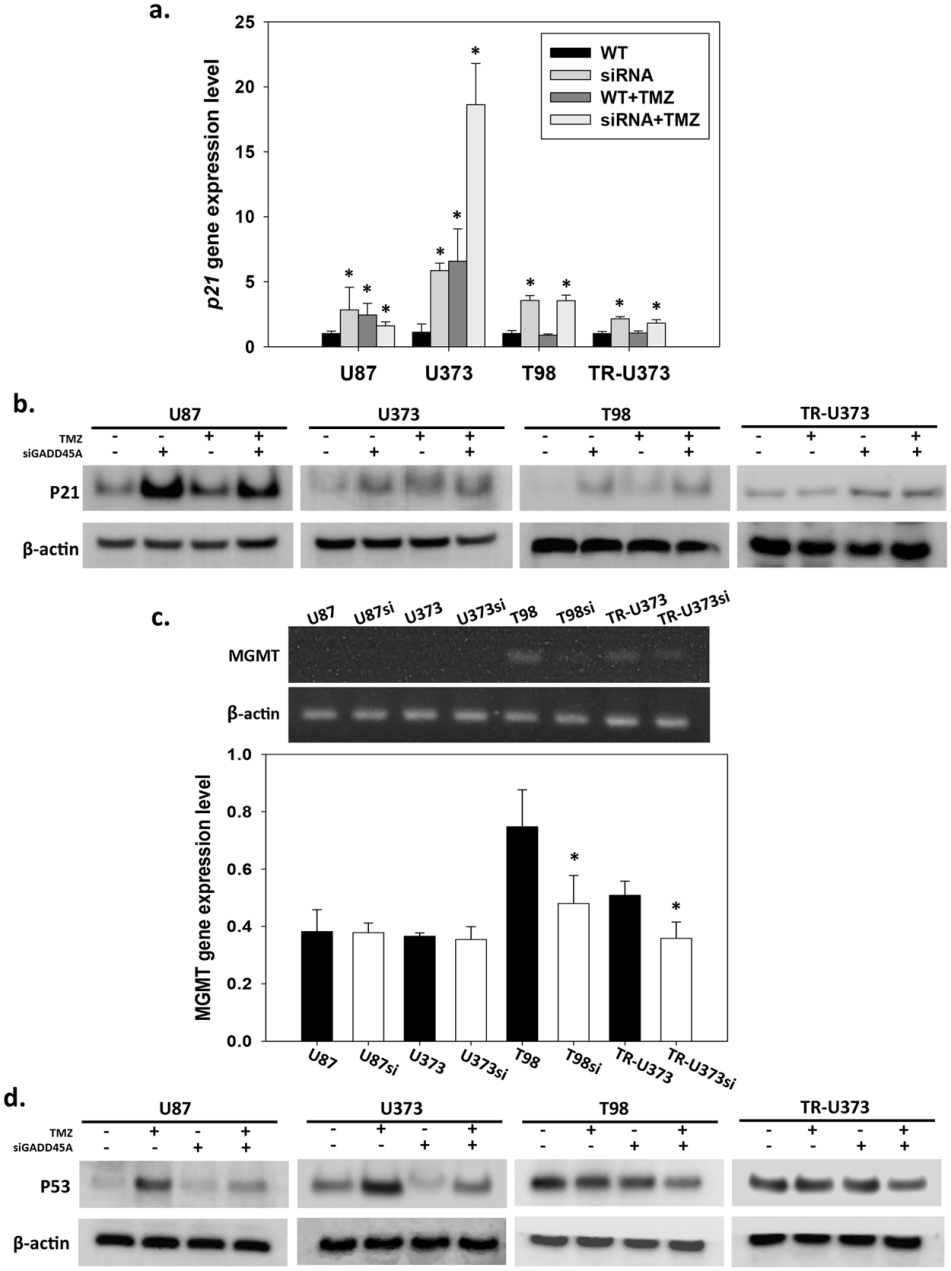
p21, MGMT and TP53 expression in U87, U373, T98 and TR-U373 cells with TMZ or GADD45A siRNA treatment. Analysis of the expression of p21 in U87, U373, TR-U373 and T98 cells treated with TMZ (25 μg/ml) or GADD45A siRNA. (**a**) Analysis of p21 gene expression in GBM cell lines by qPRC. n = 3, *p < 0.05 (**b**) Analysis of p21 protein expression in GBM cell lines by western blotting. Original blot was shown in Supplementary Fig. 8. (**c**) The *mgmt* gene expression in GBM cell lines analyzed by RT-PCR. Full-length gels were shown in Supplementary Fig. 4. (**d**) Analysis of TP53 protein expression in GBM cell lines by western blotting. Original blot was shown in Supplementary Fig. 9. n = 3, *p < 0.05.

GADD45A plays an important role in the arrest of the cell cycle in the G2/M phase in response to environmental stress^16^. As described above, we showed that TMZ-induced GADD45A led to the arrest of GBM cells in the G2/M phase; however, GADD45A^kd^ also decreased cell proliferation. To further understand the effect of GADD45A on cell growth, we examined the expression of another proliferation inhibitor, p21. Our data showed that both TMZ treatment and GADD45A^kd^ increased p21 expression and that the increase in p21 expression was more severe in U87si and U373si cells than in cells only treated with TMZ. However, p21 expression in T98 and TR-U373 cells was only elevated by GADD45A^kd^ (Fig. 7a and b). These data suggested that the G2/M arrest caused by GADD45A^kd^ was a result of increased p21 expression.

### GADD45A^kd^ was accompanied by decreased MGMT expression

The DNA repair protein MGMT is a major determinant of the resistance of tumors to various alkylating drugs. Several TMZ-resistant GBM cell lines, including T98, have high MGMT expression, and abolishing MGMT expression enhances the antitumor effect of TMZ^17, 18^. Because we showed that GADD45A^kd^ enhanced the cytotoxic effect of TMZ in various GBM cell lines, we sought to investigate MGMT expression under conditions of GADD45A^kd^. In agreement with previous findings, our data showed that MGMT was highly expressed in TMZ-resistant cell lines, including T98 and TR-U373; however, MGMT was not detected in the TMZ-sensitive U87 and U373 cell lines (Fig. 7c). Although MGMT was not detected in the TMZ-sensitive cell lines, TP53 levels were clearly decreased in U87si and U373si cells than in U87 and U373 cells, after TMZ treatment (Fig. 7d). Moreover, the MGMT level was substantially decreased in T98si and TR-U373si cells (Fig. 7c). These data suggested that the improvement in the TMZ-induced cytotoxic effect by GADD45A^kd^ in TMZ-resistant GBM cells probably have occurred as a result of decreased MGMT expression. And the protective role of GADD45A against TMZ was mediated through MGMT-independent mechanism in TMZ-sensitive GBM cells.

## Discussion

TMZ is the current standard chemotherapeutic agent used to treat GBM. However, the resistance of GBM patients to TMZ treatment leads to a poor prognosis. Therefore, it is essential to develop more effective therapeutic regimens for GBM treatment. To identify novel targets to improve the current chemotherapeutic regimens, we performed expression profiling of chemo-sensitive (U87) and resistant (T98) GBM cell lines treated with TMZ, the first-line treatment for GBM^19^. Among the fourteen genes found to be substantially regulated in TMZ-treated U87 cells, GADD45A was identified in both the cell cycle and apoptosis arrays. Therefore, we further addressed the role of GADD45A in the response of GBM cells to genotoxic stress^20^. The present study provides what is, to our knowledge, the first evidence that the GADD45A protein protects GBM cells from genotoxic stress induced by TMZ. Furthermore, GADD45A^kd^ sensitized cells, even chemo-resistant T98 and TR-U373 GBM cells, to TMZ-induced apoptosis. Thus, GADD45A probably play anti-apoptotic and protective roles in GBM.

As reported in previous studies, TMZ treatment results in a marked increase in the fraction of human GBM cells arrested in the G2/M phase^21^. Our results are consistent with findings from an earlier report showing that TMZ treatment caused an accumulation of GBM cells at the G2/M boundary. Furthermore, the inhibition of proliferation and the proportion of G2/M cells after TMZ treatment were enhanced in a dose- and time-dependent manner. The G2/M arrest was induced by DNA damage^22^. During chemotherapy, the use of alkylating agents increases the genotoxic stress-induced DNA damage^23^ and the most crucial biological response to DNA damage is cell death. Activation of cellular processes such as DNA repair and cell cycle arrest regulates this biological response immediately after DNA damage^24, 25^. The tumor-suppressor TP53 and its downstream molecules have been shown to play an important role in the cellular response to a variety of DNA-damaging agents^26^. The first well-defined TP53 downstream gene, GADD45A has been shown to be immediately induced by DNA damage caused by irradiation and genotoxic drugs, such as cisplatin and 5-fluorouracil^27–29^. Here, we showed that a high level of GADD45A expression can also be induced by TMZ treatment.

GADD45A is a stress-induced protein that has been found to interact with other cellular molecules implicated in the maintenance of genomic stability^30^, the control of the cell cycle at the G2/M checkpoint^31^, apoptosis^32, 33^ and DNA repair^34, 35^. Thus, GADD45A is involved in the pathogenesis of many types of human cancers. In a previous study, we have shown that GADD45A expression is associated with GBM malignancy^36^. Here, we further showed that GADD45A is highly expressed in drug-sensitive GBM cells after TMZ treatment, and GADD45A^kd^ substantially inhibited proliferation and increased apoptosis in response to TMZ treatment even in drug-resistant GBM cells. In addition, although GADD45A expression was not affected by TMZ in TR-U373 cells, it was originally expressed at a higher level in TR-U373 cells compared with U373 cells. All of these findings suggest that GADD45A plays a protective role in GBM.

On the basis of the protective function described above, several cellular molecules, including p21 and proliferating cell nuclear antigen (PCNA), which are involved in genomic stability and have been shown to interact with GADD45A, were also detected in this study. PCNA is a DNA repair protein that stimulates DNA excision repair^37^. When cells are exposed to a genotoxic environment, GADD45A is activated which led to enforcement G2/M checkpoint thereby providing time for DNA repair^38^; further, the activated GADD45A promotes DNA repair via interaction with PCNA and apurinic/apyrimidinic endonuclease (APE1) as a chemopreventive mechanism^39^. Here, we showed that PCNA was elevated together with GADD45A by TMZ, thus suggesting that GADD45A promoted cell survival and protected cells from TMZ treatment through interaction with PCNA to enhance DNA repair. Furthermore, the ubiquitination of PCNA is a relevant feature in PCNA-dependent DNA repair and the stable expression of p21 causes a substantial decrease in UV-induced ubiquitinated PCNA and in PCNA-dependent repair^40^. Here, we also showed that p21 expression was elevated by GADD45A^kd^. Together, our results suggested that GADD45A may promote PCNA-dependent DNA repair through down-regulation of p21 as a protective mechanism against TMZ treatment in GBM cells. Furthermore, our results showed that GADD45A^kd^ inhibited GBM cell proliferation, and this phenomenon was also observed in pancreatic cancer, melanoma and T cells^41–44^. The elevation of the proliferation inhibitor p21 by GADD45A^kd^ may be involved in the inhibition of proliferation.

Similarly to our results, a previous study has shown that GADD45-deficient mice exhibit greater genomic instability^30^ and a substantial defect in global genomic repair of embryonic fibroblasts^34^. Down-regulation of GADD45A decreases the survival of colon cancer^45^, hematopoietic cells^20, 46^, melanoma^44^ and epidermal cells^47^ after exposure to UV radiation, certain chemotherapeutic drugs or oxidative stress. Furthermore, GADD45A deficiency sensitizes brain epithelial cancer cells to ionizing radiation (IR) and extends survival in IR-treated mice with brain tumors^48^. However, some studies have shown the opposite result, in which GADD45A enhances irradiation-induced apoptosis in medulloblastoma^49^, oral squamous cell carcinoma^50^ and keratinocytes^50^. GADD45A has been reported to be involved in both apoptosis and cell survival through different cellular pathways; through the p38-JNK MAPK pathway, GADD45A acts as a pro-apoptotic protein that induces apoptosis and cell cycle arrest^51^. In contrast, GADD45A protects cells from genotoxic stress-induced cell death through activation of the p38-NF-κB survival pathway^20^. These different functions of the GADD45A protein are mediated by protein-protein interactions and depend on the biological setting, including the cell type, developmental stage, and stress/stimulus^52^. The experiments performed in this study indicated that GBM cells with GADD45A^kd^ exhibited an increased rate of cell death in response to TMZ treatment. This study demonstrated that, under TMZ-induced genotoxic stress, GADD45A mediates the survival pathway in GBM cells.

Notably, TMZ sensitivity was increased by GADD45A^kd^ not only in TMZ-sensitive GBM cells but also in TMZ-resistant GBM cells. A growing body of evidence indicates that MGMT plays an important role in resistance to alkylating agents in GBM cells^53^. MGMT can repair O^6^-alkylguanine adducts and directly reverse methylation damage induced by these chemotherapy drugs^18^. Here, we showed that cell proliferation and the cell cycle were not affected by TMZ in TMZ-resistant T98 and TR-U373 cells; however, these two cell lines became sensitive to TMZ with GADD45A^kd^, thus suggesting that GADD45A may affect the original chemo-resistant system in T98 and TR-U373 cells. Here, we showed that MGMT was highly expressed in TMZ-resistant T98 and TR-U373 cells, but it was decreased by GADD45A^kd^. MGMT is regulated by TP53 in several human cancers. Wild-type TP53 inhibits MGMT expression by preventing the binding of the Sp1 transcription factor to the cognate cis-elements in the MGMT promoter^54^. In contrast, mutant TP53 positively regulates MGMT expression in glioma cells^55^. It has been shown that T98 and U373 cells have mutant TP53. Here, we further showed that GADD45A^kd^ down-regulated TP53 expression in both T98 and TR-U373 cells. Similarly to our findings, although GADD45A is recognized as a conventional downstream gene of TP53, it also plays a role as upstream effector in TP53 phosphorylation and stabilization^56, 57^. Collectively, these findings suggested that GADD45A may positively modulate MGMT expression through the mutant TP53 pathway and thus regulate chemo-sensitivity in MGMT-proficient/TMZ-resistant GBM cells. However, our results showed that GADD45A^kd^ also enhanced chemo-sensitivity in TMZ-sensitive U87 and U373 cells which expressed an extremely low level of MGMT (undetectable in this study). As shown by Hegi *et al*., although the benefit from TMZ is significantly associated with methylated MGMT promoter, some GBM patients with methylated tumor still have short survival rate^58^. These exceptions imply that the existence of additional MGMT-independent mechanisms involved in TMZ response. Aware that TP53 plays an important role in response to DNA damage, the effect of TP53 for response to DNA alkylating agents has been investigated. Several studies show that loss of TP53 function significantly increase the chemo-sensitivity of GBM cells to BCNU and TMZ, no matter whether the cells express wild-type or mutant TP53^55, 59–61^. Here we showed that underlying the process of GADD45A^kd^ enhanced chemo-sensitivity, the TP53 expression was decreased not only in wild-type (U87) but also in mutant (U373, T98) TP53-expressing GBM cells. Similar to our finding, GADD45A contributes to TP53 stabilization in response to UVB-induced DNA damage in embryonic fibroblasts derived from TP53 wild-type mice^56^. In prostate cancer cells, knockdown of GADD45A abrogates serine 15 phosphorylation of mutant TP53, leading to a decrease of TP53 stability^62^. These evidences indicate that GADD45A participates as part of the positive feedback signal in TP53 pathway activation. Collectively, our data suggested that in MGMT-deficient GBM cell lines, GADD45A still can protect cells from TMZ-induced genotoxicity through MGMT-independent/TP53 pathway.

Together, here we showed that GADD45A protein protects different GBM cell lines, which express different level of chemo-sensitivity and TP53 status, from TMZ induced genotoxicity. This protective function of GADD45A probably through TP53-dependent and MGMT-dependent pathway in TMZ-sensitive and TMZ-resistant GBM, respectively. Although the mechanism involved remains unclear, our results suggest that the primary mechanism by which GADD45A plays a protective role against TMZ in GBM cells and the regulation between GADD45A and MGMT are important issues that should be clarified. This study clearly showed that the GADD45A protein protects GBM cells from genotoxic stress induced by TMZ; therefore, GADD45A has protective and anti-apoptotic functions in GBM. Systemic chemotherapy is an important part of the current treatment approach for patients with GBM^63^. Given that GADD45A is not a frequent target of mutations in cancers and that no reports of GADD45A mutations in GBM have been published to date^29^, this protective role of GADD45A against TMZ may be applied to the development of additional local therapeutics for use along with systemic TMZ treatment in GBM patients and may provide a new therapeutic prospect for GBM treatment.

## Material and Method

### Chemicals and medium

The DMEM medium (with HEPES) was purchased from GIBCO (Gaithersburg, MD). Fetal bovine serum (FBS) and antibiotics were obtained from Hyclone (Logan, UT). Other chemicals and supplies were obtained from Sigma, (St. Louis, MO) unless otherwise indicated.

### Cell culture and viability assay

Human glioblastoma U87, U373 and T98 cell lines were obtained from American Type Culture Collection (ATCC: Rockville, MD, USA). It has been shown that the DNA profile of U87 cell line is different from that of the original cells^64^, but the gene expression profile indicates that the ATCC’s U87 cell line is of CNS origin and probably be GBM cell line from unknown patient. Thus, U87 is still used as cell model of GBM. To ensure the integrity of our research results, the U87 cell lines were used less than 35 passages from the original obtained from ATCC, and were authenticated by STR profiling (Mission Biotech, Taiwan). Cells were maintained in DMEM with 10% FBS at 37 °C in a humidified 5% CO_2_ atmosphere. To develop TMZ-resistant (TR) cells, U373 cell line was initially cultured in the presence of 6.25 µM of TMZ. The concentration of TMZ was increased by 2-fold for every two weeks (about four passages) until it reached 50 µM (maximum concentration) for over a month. To select the stable TR-U373 clone, the trypan blue exclusion method was used to investigate cell viability and proliferation, and the TR-U373 cell was used when it’s growth rate approximate to normal U373.

### Cell growth curve

Cell growth curve was assayed using 3-(4,5-dimethylthiazol)-2,5- diphenyltetrazilium bromide (MTT) dye (Sigma) as previously described^28^. Briefly, 2.5 ml cells (2.4 × 10^4^/ml; DMEM; log phase) were plated into 96-well plates and incubated overnight to allow attachment. The cells were then treated with a serum-free medium and different concentrations of TMZ. After 0, 1, 2, 3, 4 and 5 days, 20 μg MTT solution (500 μg/ml) was added to each well and incubated for 4 hours to form the formazan crystals. Subsequently, the cells were solubilized with dimethysulfoxide (DMSO). The 570 nm absorbance was used to determine the cell activity by a multiscan reader (Dynatech, VA, USA).

### Cell cycle analysis

The human U87 GBM, human U373 GBM-astrocytoma-epithelial-like cell, human T98 GBM, rat C6 glioma, human HaCaT keratinocyte and human Z172 cervical epithelium cancer cells were treated with or without different concentrations of TMZ for various time periods and were collected through trypsinization, fixed in 70% ethanol at 4 °C overnight, centrifuged at 500 g for 5 minutes. The cell pellets were washed with ice-cold PBS. In the dark, the cells were labeled with 1 ml PI solution (including 20 μg/ml PI, 0.1% TritonX-100, 0.2 mg/ml RNase A, and PBS) for 30 minutes at room temperature. The cells were then assayed by flow cytometry (FACSCan; Becton Dickinson), and at least 10,000 cells were counted in each sample.

### Western blotting analysis

For western blotting analysis of the BNIP3, GADD45A and p21 gene products, the total protein (15 μg) for TMZ treated-U87 and untreated-U87 control cells was electrophoresed in 4–12% polyacrylamide gel. The cell suspension was briefly washed 3 times with PBS at pH 7.4 and homogenized in a lysis buffer (PBS with 10 mM EDTA, 1 mM PMSF, pH 7.4). Nuclei and undisrupted cells were removed by centrifugation and the supernatant was lyophilized at −40 °C. Lyophilized protein was rehydrated using 20 mM HEPES and 1 mM PMSF at pH 7.4. The protein was quantified (5 μg or 2.5 μg) and electrophoresed in a polyacrylamide gel. After electrophoresis, a nitrocellulose membrane (Hybond, Amershan) was placed under the gel while 3 absorbent papers were placed on each side of gel. The membrane and absorbent papers were pre-wetted with TBE buffer (0.1 M Tris, 0.1 M boric acid, 2 mM EDTA). The resolved proteins were electrophoretically transferred to nitrocellulose membrane in a BIO-RAD semi-dry transfer cell at 1 mA/cm^2^ of gel for 2 hours. After transfer the protein, the blots were soaked in TTBS buffer (10 mM Tris, pH 8.0, 150 mM NaCl, 0.05% Tween 20) with 15 ml 5% BSA for 1 hour and were subsequently incubated with primary monoclonal antibody against BNIP3 (Abcam, Cambridge, UK; diluted 1:1000), GADD45A (Millipore, MA; diluted 1:2000), p21 (Abcam, Cambridge, UK; diluted 1:2000) and TP53 (Abcam, Cambridge, UK; diluted 1:2000) gene products while goat anti-mouse IgG (Sigma) was used as secondary antibody in 15 ml TTBS buffer containing 1% BSA at 4 °C overnight. Following antibody incubation, the blot was washed three times with 15 ml TTBS buffer, each time for 15 minutes, and incubated with secondary antibody (sheep anti-goat IgG, HRP conjugate 1:10000, Amershan) in 15 ml TTBS buffer containing 1% BSA. After washing three times with TTBS buffer, the blots were further washed with 15 ml TBS (TTBS without Tween 20) to wash away Tween 20. Finally, the blots were air-dried and developed in DAB substrate solution. The DAB substrate solution was prepared by mixing 4.5 ml of 0.5 mg/ml 3,5-diaminobenzide (DAB) in PBS, pH 7.4 with 0.5 ml of 0.3% NiCl_2_ in H_2_O and 5 μl of 30% H_2_O_2_. Finally, the developed blots were washed in H_2_O to stop the reaction. The bands were analyzed using the IS-1000 Digital Imaging System (Alpha Innotech Coperation).

### RNA isolation and microarray hybridization

The total RNA from U87 cells treated and untreated with TMZ were performed. Briefly, total of 5 × 10^6^ cells were collected and processed for total RNA with a commercial kit (TRIzol Plus RNA Purification System, Invitrogen, USA) according to the manufacturer’s protocol. The quality of RNA was estimated by the OD 260/280 ratio and 28 S/18 S ratio on argarose gel electrophoresis. Fluorescent labeled cDNA probes were made from 5 μg aliquots of total RNA samples by oligo (dT)-primer reverse transcription using Superscript II reverse transcriptase (Gibco, USA). Fluorescent nucleotide Cy5- and Cy3-dUTPs (GE Healthcare, UK) were used to label the cDNA of the experimental and reference tissue, respectively. The probes were purified by QIA Purification kit (Qiagen, CA). The blocking reagents, 50% formamide, 10X SSC (sodium saline citrate, Sigma-Aldrich, USA), 0.2% SDS (sodium dodecyl sulfate, Sigma-Aldrich, USA), 20 μg of poly (dA) polymer (GE Healthcare, UK), and 20 μg of human COT-1 DNA (Gibco, USA), were added to the probes. The purified and concentrated fluorescent-labeled cDNA from reference and experimental samples were mixed, and a hybridization mixture was prepared in a final volume of 15 μg containing 3.4X SSC and 0.3% SDS. The hybridization mixture was incubated at 95 °C for 2 min then at room temperature for 30 min, and mounted on the microarray glass. After placing a coverslip on the slide, hybridization was performed at 65 °C for 16 hr in a Hybridization Cassette (Agilent, USA). The slides were washed with 0.6X SSC containing 0.03% SDS for 10 min and 0.05X SSC for 5 min twice at 42 °C. The slides were dried and subjected to image processing.

### siRNA transfection

The transfection was performed by using the DhamaFECT1 siRNA transfection reagent (Dharmacon, USA) according to the manufacturer’s protocol. Briefly, 3.0 × 10^5^ cells were added to each well of a 6-cm^2^ culture plate containing complete DMEM with 10% FBS and incubated overnight for cell attachment. Then medium was removed and fresh DMEM medium without antibiotics was added. The transfection reagent was mixed with GADD45A-siRNA or ddH_2_O and incubated for 20 min at room temperature. The GADD45A-siRNA-treated cells and controls cells were treated TMZ or DMSO after 24 hours, and the cells were harvested after 4 days drug treatment for further analysis. During the experiment, the GADD45A-siRNA was supplemented once every 3 days.

### RT-PCR assay

Gene expression level was determined by one-step RT-PCR following the manufacturer’s instructions (SuperScript, invitrogen, USA). In brief, cells treatment and RNA extractions were the same as described above. As shown in Table 2, it demonstrated that specific primer and probe sequences for RT-PCR assay of BNIP3, GADD45A and p21, genes. The GAPDH and β-actin gene played a housekeeping and internal control role. All experimentations were performed independently in triplicate. Image analysis and quantification were performed by using Phoretix 1D (totallab, USA).

**Table 2:** Primer list.

Annotation	Primer sequence	Cycle	T (°C)	Length (bp)
BNIP3	5′-CCACCTCGCTCGCAGACACCAC-3′	32	61	316
	5′-GAGAGCAGCAGAGATGGAAGGAAAAC-3′			
GADD45A	5′-TCAGCGCACGATCACTGTC-3′	32	55	372
	5′-CCATTGATCCATGTAGCGAC-3′			
p21	5′-CGACTGTGATGCGCTAATGG-3′	35	55	249
	5′-AGTGACAGGTCCACATGGTC-3′			
MGMT	5′-GTGATTTCTTACCAGCAATTAGCA-3′	32	55	127
	5′-CTGCTGCAGAGACCACTCTGTG-3′			
β-actin	5′-TGGAATCCTGTGGCATCCATGAAAC-3′	25	51	348
	5′-TAAAACGCAGCTCAGTAACAGTCCG-3′			

### Giemsa stain

The cells were washed by PBS and fixed with 0.5 ml fixation solution (methanol (Merck, Germany): acetic acid (Merck, Germany) = 3:1) for 30 minutes. The cells were immersed with freshly prepared Giemsa’s buffer (6‰ Na_2_HPO_4_ (American Biorganics, INC), 5‰ KH_2_PO_4_ (American Biorganics, INC), pH 7) for 10 minutes, then stained by the Giemsa’s azur eosin emthylene blue solution (Merck, Germany) for 45 minutes. Rinse cells with the Giemsa’s buffer several times, and rapidly dipped in 5% acetic acid (Merck, Germany). Cells were dehydrated by 100% alcohol (Merck, Germany) and xylene (Kanto Chemical, CO). After air- drying, cells morphology was observed by light microscopy.

### Real-time qPCR

Total RNA was reverse transcribed into cDNA using High Capacity cDNA Reverse Transcription Kit (Applied Biosystems, USA) according to the manufacturer’s instruction. Quantitative amplification of the cDNA templates was carried out using a SYBR® Green PCR Master Mix (Applied Biosystems, USA) in a StepOne™ Real-Time PCR System (Applied Biosystems, USA). Amplification of *β*-actin mRNA was used as the loading control. The absolute levels of the mRNA were normalized by *β*-actin mRNA content. Primers were listed in Table 2.

### Apoptosis assay

Cell apoptosis was detected using an annexin V/PI detection assay. Briefly, cells were treated with DMSO or 25 μg/ml TMZ. After 96 hours, the cells were stained by annexin V-FITC apoptosis detection kit (Strong Biotech Corp, TW) and analyzed by flow cytometry (FACSCan, Becton Dickinson). The FlowJo software (Treestar, Inc., CA) was used to analyze the percentage of cells in four populations, including FITC^−^/PI^−^ (living cells), FITC^+^/PI^−^ (early apoptotic cells), FITC^+^/PI^+^ (late apoptotic cells) and FITC^−^/PI^+^ (necrotic cells). The fold change of apoptosis was calculated as the ratio of all apoptotic cells (including early and late apoptotic cells) under different conditions to control.

### Statistical analyses

Statistical analyses were performed with the Mann Whitney U test. The significance of differences among values was *p < 0.05. Data in the text were represented as mean ± SD.

## Electronic supplementary material

Supplementary information
