# Supplementary material for: GADD45A plays a protective role against temozolomide treatment in glioblastoma cells
Source: Sci Rep. 2017 Aug 18;7:8814. doi: 10.1038/s41598-017-06851-3 (PMC5562912; doi:10.1038/s41598-017-06851-3)
Supplement: Supplementary file 1 — Supplementary information [file 41598_2017_6851_MOESM1_ESM.pdf]

Full title:

GADD45A plays a protective role against temozolomide treatment in glioblastoma cells

Short title:

GADD45A protects glioblastoma cells from temozolomide treatment

Authors:

Hsiao-Han Wang[1], Tsuey-Yu Chang[2], Wei-Chen Lin[2], Kuo-Chen Wei[3*], Jyh-Wei Shin[2*]

Affiliations:

[1] Institute of Basic Medical Sciences, College of Medicine, National Cheng Kung University, Tainan, Taiwan; [2] Department of Parasitology, College of Medicine, National Cheng Kung University, Tainan, Taiwan; [3] Departments of Neurosurgery, Chang Gung Memorial Hospital, College of Medicine, Chang Gung University, Taoyuan, Taiwan.

Correspondence to: Jyh-Wei Shin, Department of Parasitology, National Chung Kung University, No.1, University Road, Tainan, Taiwan, R.O.C. Tel: +88662353535, ext 5611, Fax: +88662387614, e-mail: hippo@mail.ncku.edu.tw and Kuo-Chen Wei, Department of Neurosurgery, Chang Gung Memorial Hospital, Chang Gung University College of Medicine, No. 5, Fu Shin Street, Kweishan, Taoyuan, Taiwan, R.O.C. Tel: +88633281200, ext. 2412, Fax: +88633285818, e-mail: kuochenwei@adm.cgmh.org.tw

Supplementary information

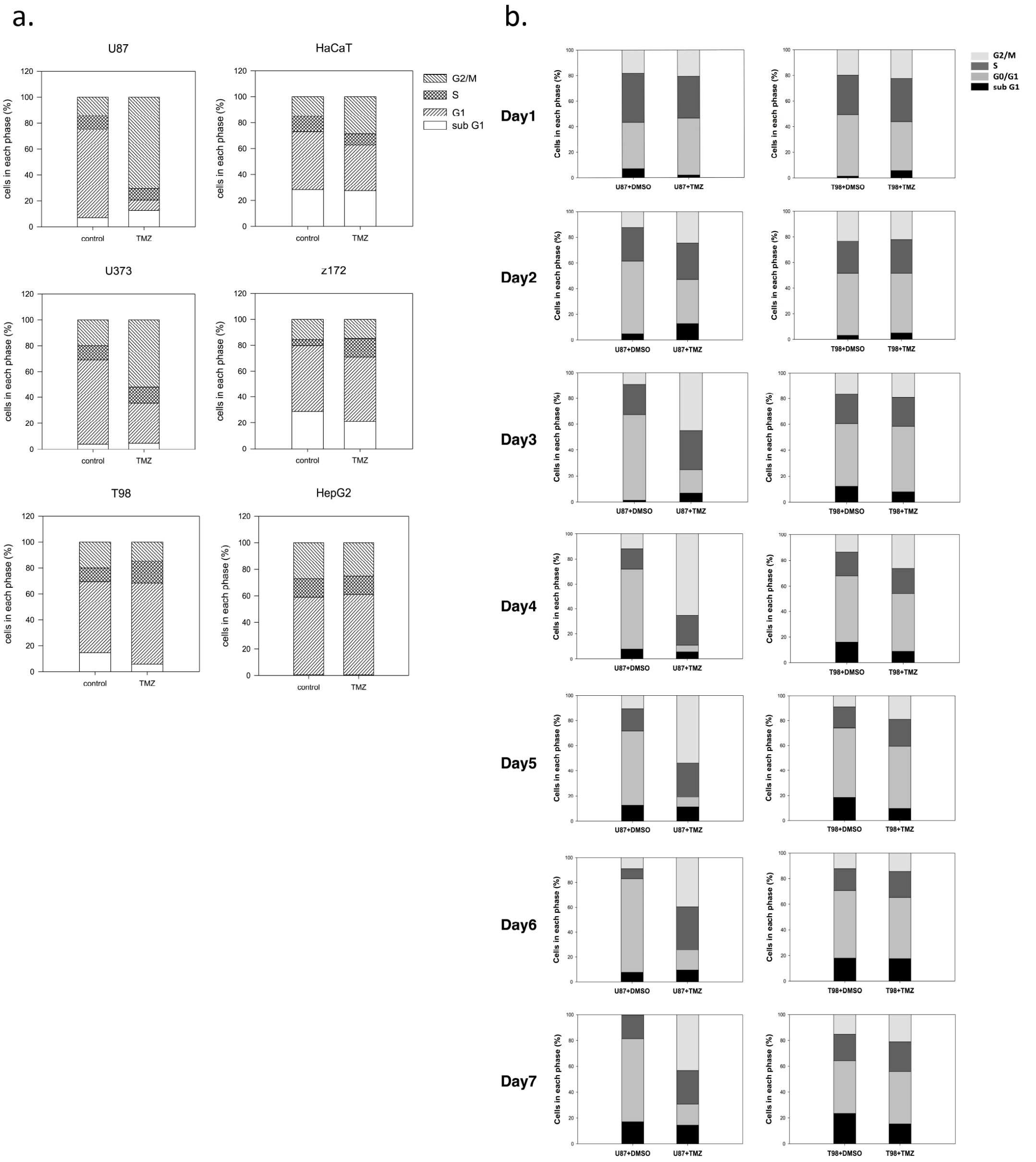

Supplementary Figure 1. Cell cycle progression after TMZ treatment of human GBM or non-GBM cell lines analysis by FACS. Bar charts illustrating the proportion of cells at different stages in the cell cycle derived from the decomposition of flow cytometry histograms. **(a)** Different human cells were treated with 25μg/ml TMZ for 4 days. U87, U373, T98: GBM cell lines; HaCaT: keratinocyte cell line; Z172: cervical epithelial cell line; Hep G2: hepatocellular carcinoma cell line. **(b)** U87 and T98 cells were treated with 25μg/ml TMZ for different time frames: day 1 to

day 7.

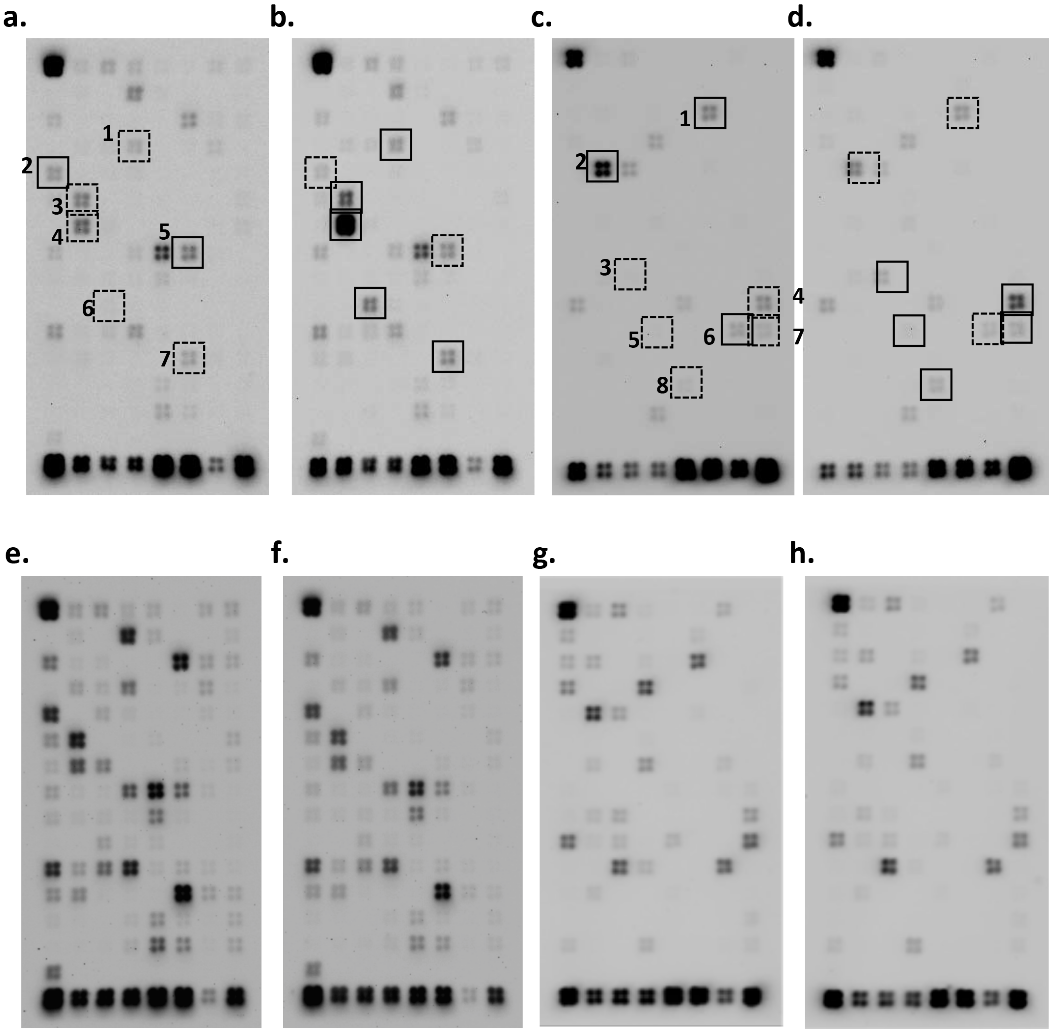

Supplementary Figure 2. Cell cycle and apoptosis related genes expression profile of U87 and T98 cells treated with or without TMZ. GEArray membranes containing human cell cycle or apoptosis gene-specific cDNA fragments were hybridized with labeled cDNA probes synthesized from total RNA derived from U87 and T98 cells treated with or without TMZ (25μg/ml). Human cell cycle microarray derived from U87 cells treated with DMSO only **(a)** and TMZ **(b)**. Human apoptosis microarray derived from U87 cells treated with DMSO only **(c)** and TMZ **(d)**. Human cell cycle microarray derived from T98 cells treated with DMSO only **(e)** and TMZ

(f). Human apoptosis microarray derived from T98 cells treated with DMSO only **(g)** and TMZ **(h)**.

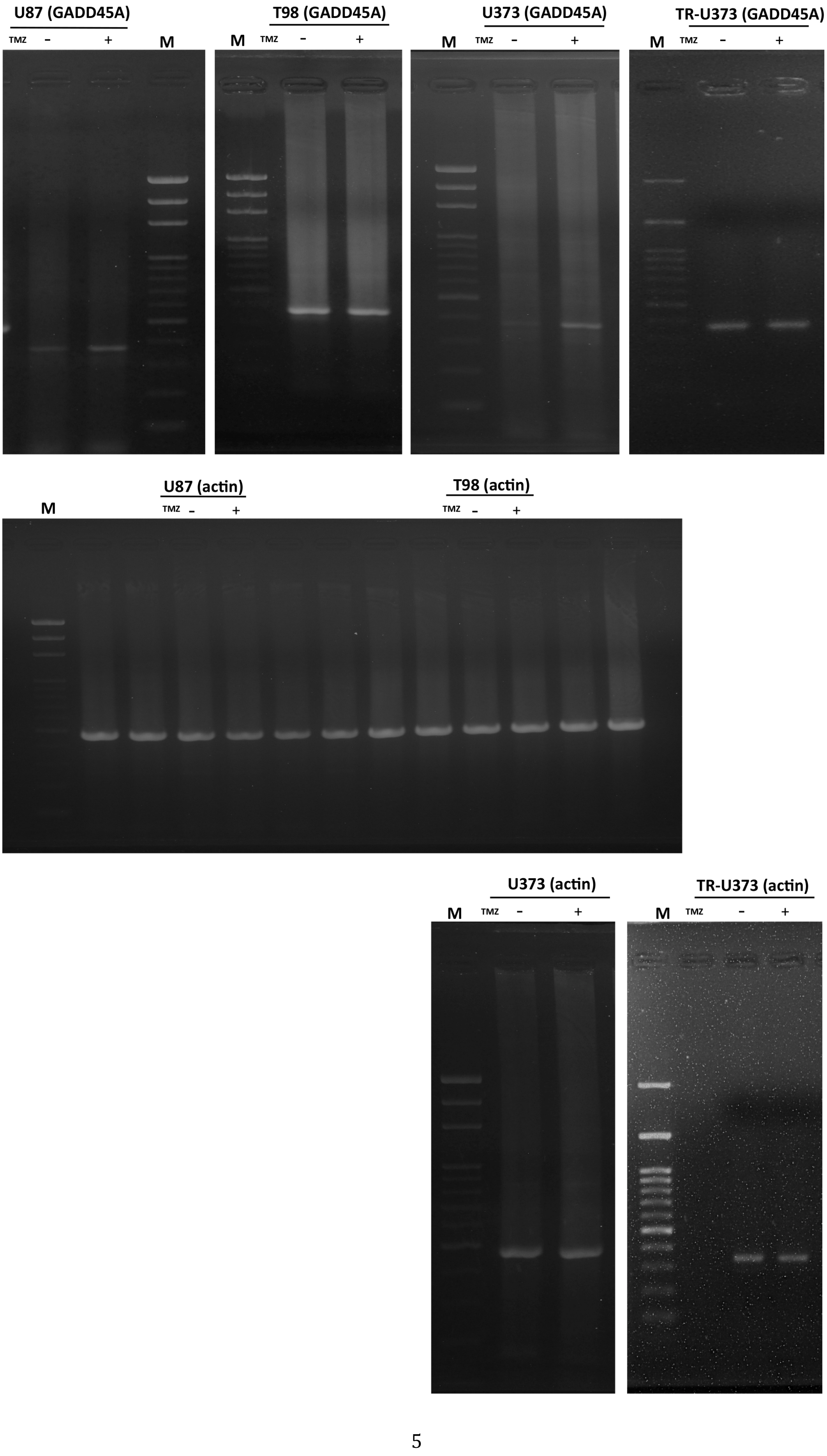
U87 (GADD45A)
TMZ - + M
T98 (GADD45A)
M TMZ - +
U373 (GADD45A)
M TMZ - +
TR-U373 (GADD45A)
M TMZ - +
U87 (actin)
M
TMZ - +
T98 (actin)
TMZ - +
U373 (actin)
M TMZ - +
TR-U373 (actin)
M TMZ - +

Supplementary Figure 3. The full-length gels of Figure 3c, d, e, f. The GADD45A expression affected by TMZ in U87, U373, T98 and TR-U373 cells.

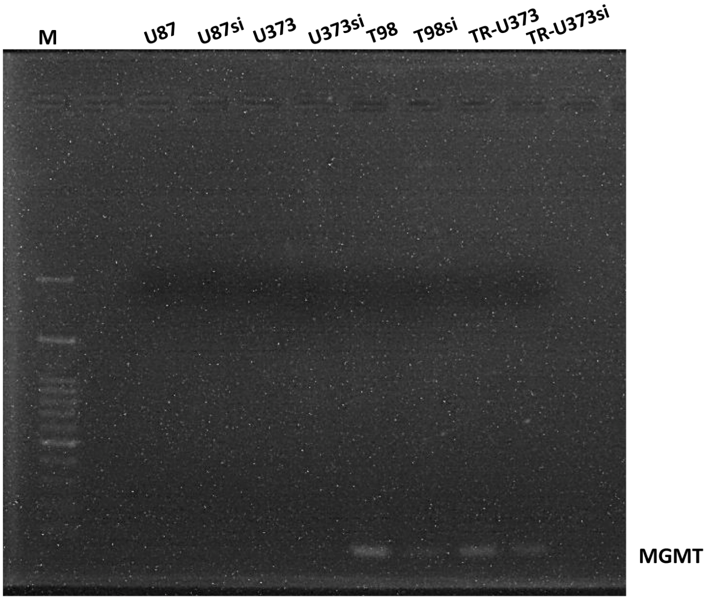

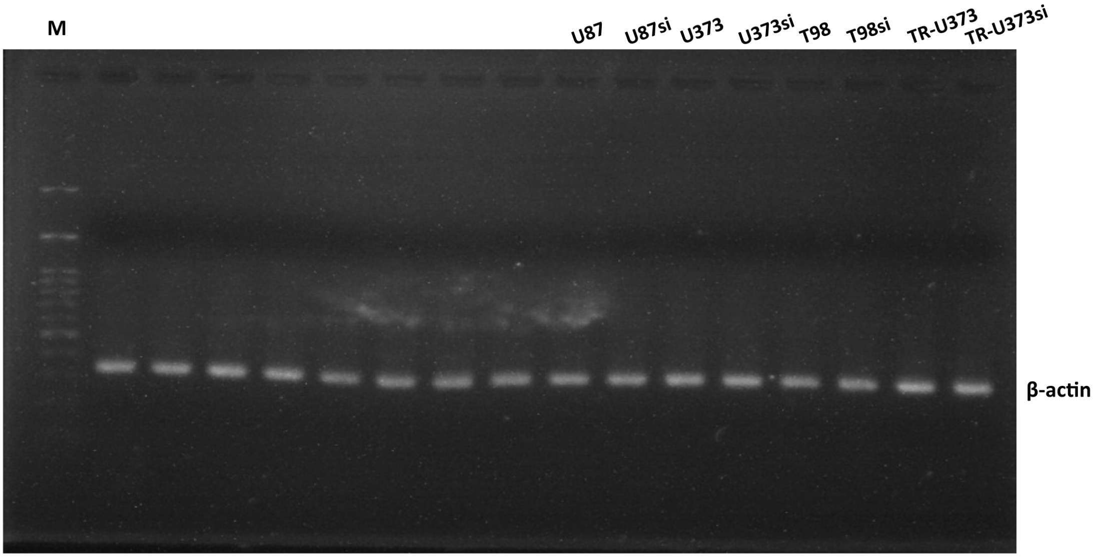

Supplementary Figure 4. The full-length gels of Figure 7c. The MGMT expression affected by GADD45A^{kd} in U87, U373, T98 and TR-U373 cells.

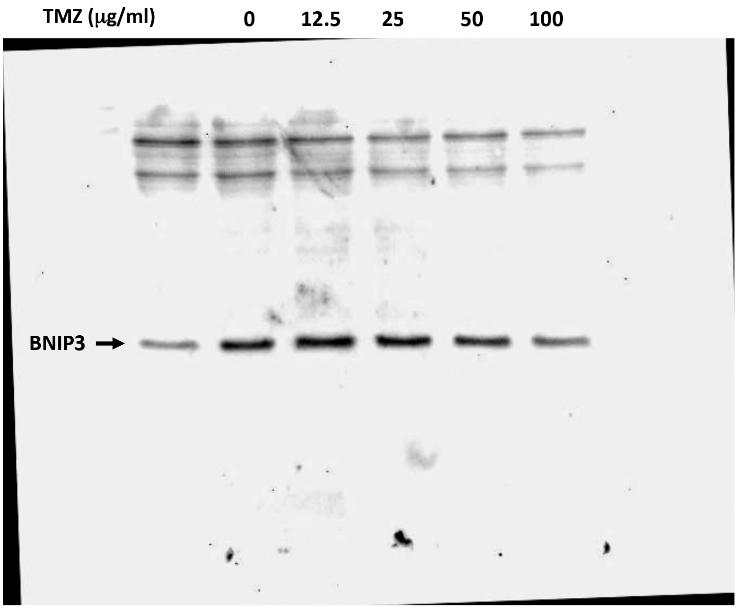

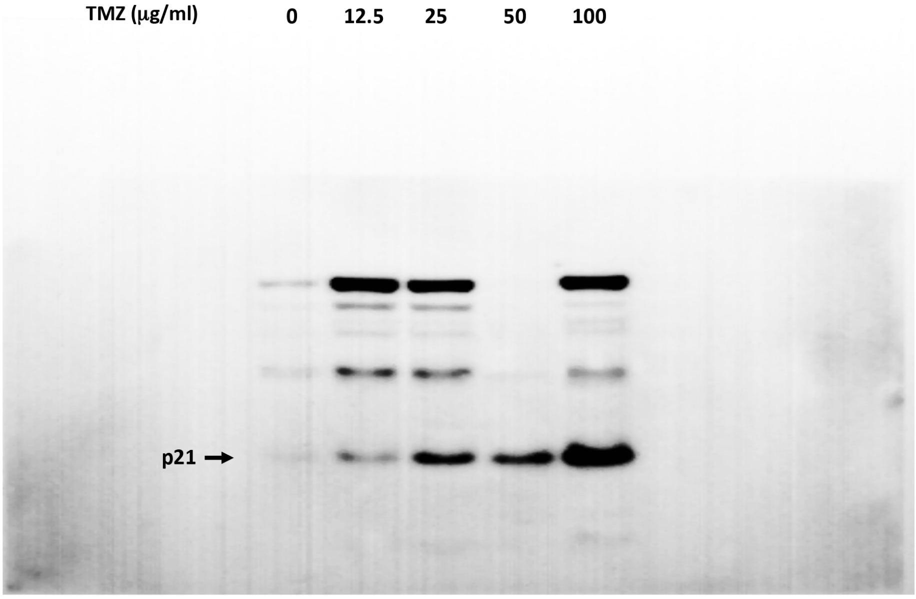

Supplementary Figure 5. The full-length blot of Figure 3b. The BNIP3 and P21 expression affected by different TMZ dose in U87, U373, T98 and TR-U373 cells.

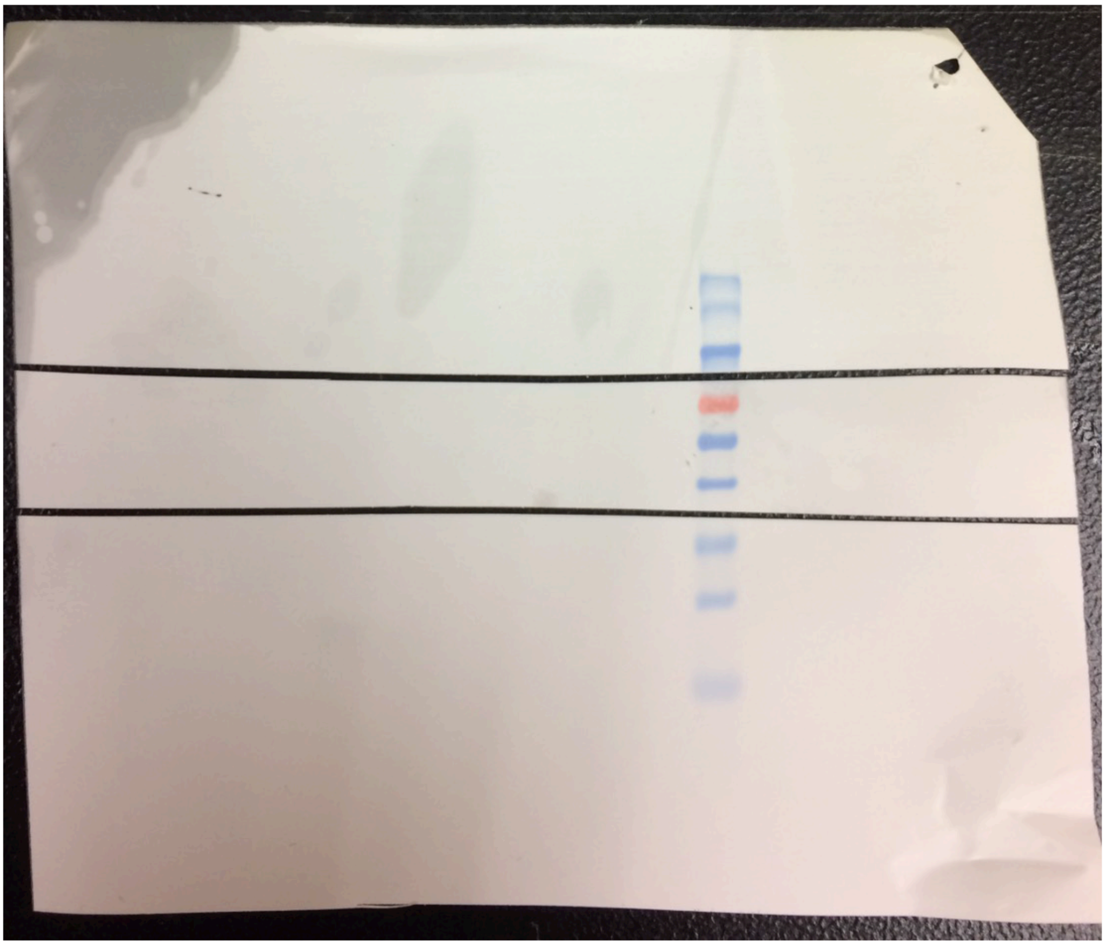

Supplementary Figure 6. The western blot membrane cropped by specific molecular weight.

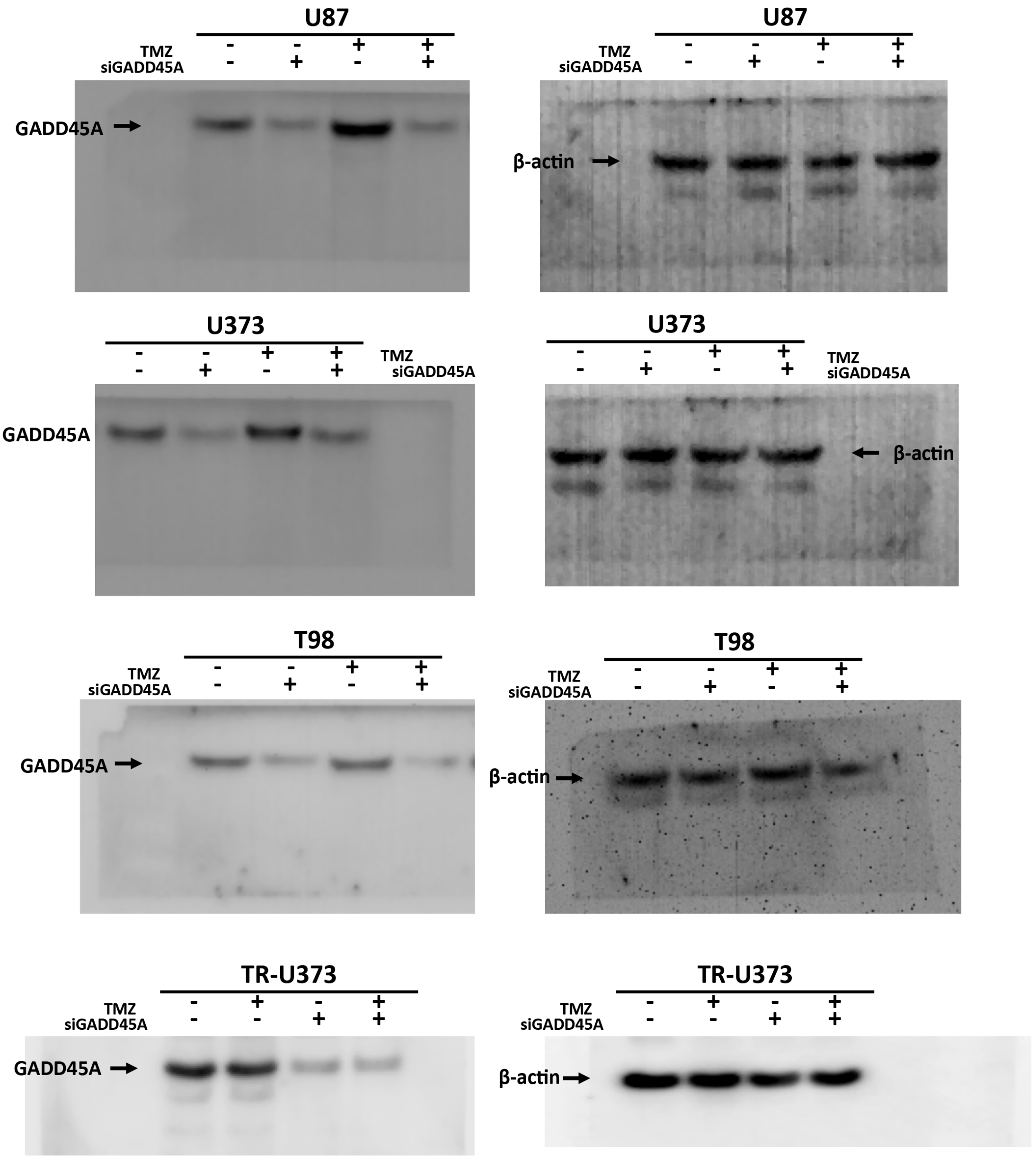

Supplementary Figure 7. The original western blot of Figure 4b. The GADD45A expression affected by TMZ and GADD45A^{kd} in U87, U373, T98 and TR-U373 cells.

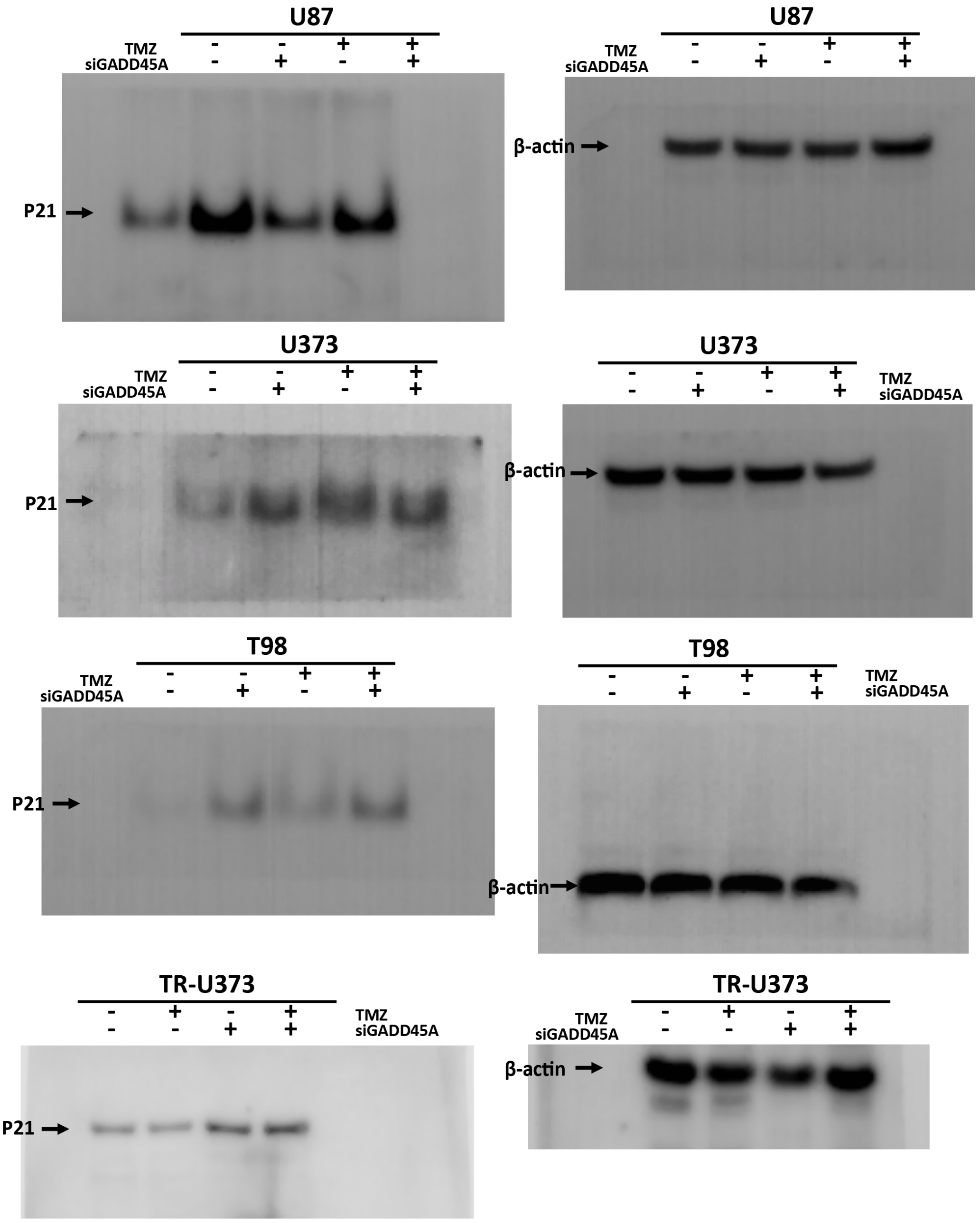

Supplementary Figure 8. The original western blot of Figure 7b. The P21 expression affected by TMZ and GADD45A^{kd} in U87, U373, T98 and TR-U373 cells.

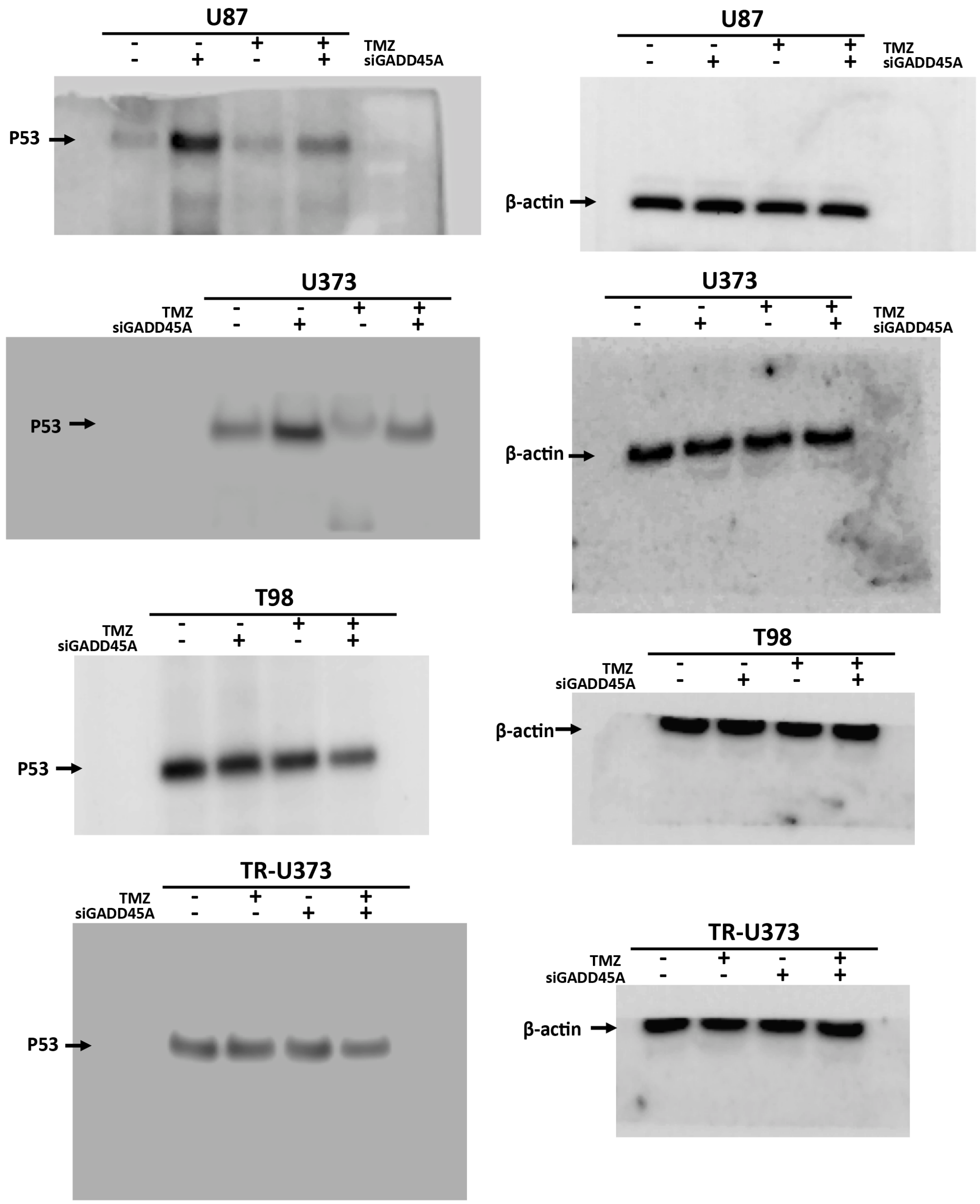

Supplementary Figure 9. The original western blot of Figure 7d. The TP53 expression affected by TMZ and GADD45A^{kd} in U87, U373, T98 and TR-U373 cells.
